# Identifying a Polymorphic ‘Switch’ That Influences miRNAs' Regulation of a Myasthenia Gravis Risk Pathway

**DOI:** 10.1371/journal.pone.0104827

**Published:** 2014-08-12

**Authors:** Lili Yang, Jianjian Wang, Xuesong Sun, Yuze Cao, Shangwei Ning, Huixue Zhang, Lixia Chen, Ronghong Li, Qinghua Tian, Lihua Wang, Weizhi Wang, Xia Li

**Affiliations:** 1 Department of Neurology, The Second Affiliated Hospital, Harbin Medical University, Harbin, Heilongjiang Province, China; 2 College of Bioinformatics Science and Technology, Harbin Medical University, Harbin, Heilongjiang Province, China; Sudbury Regional Hospital, Canada

## Abstract

The significant roles of genetic variants in myasthenia gravis (MG) pathogenesis have been demonstrated in many studies, and recently it has been revealed that aberrant level/regulation of microRNAs (miRNAs) might contribute to the initiation and progression of MG. However, the dysfunction of miRNA associated with single nucleotide polymorphisms (miRSNPs) has not been well investigated in MG. In this study, we created a contemporary catalog of 89 MG risk genes via manual literature-mining. Based on this risk gene catalog, we obtained 18 MG risk pathways. Furthermore, we identified 93 miRNAs that target MG risk pathways and revealed the miRSNPs ‘switches’ in miRNA regulation in the MG risk pathways by integrating the database information of miRSNPs. We also constructed a miRNA-mediated SNP switching pathway network (MSSPN) to intuitively analyze miRNA regulation of MG risk pathways and the relationship of the polymorphism ‘switch’ with these changes in regulation. Moreover, we carried out in-depth dissection on the correlation between hsa05200 (pathway in cancer) and MG development, and elaborated the significance of 4 high-risk genes. By network analysis and literature mining, we proposed a potential mechanism of miRSNPs→gene→pathway effects on MG pathogenesis, especially for rs28457673 (miR-15/16/195/424/497 family)→*IGF1R*→hsa05200 (pathway in cancer). Therefore, our studies have revealed a functional role for genetic modulators in MG pathogenesis at a systemic level, which could be informative for further miRNA and miRSNPs studies in MG.

## Introduction

Myasthenia gravis (MG) is a neuromuscular autoimmune disease characterized by muscle weakness that fluctuates, worsening with exertion, and improving with rest [1]. Although MG is usually sporadic, familial clustering has been reported with MG, and about 4% of MG patients have a positive family history [2]. Genetic factors have been suggested for autoimmune MG since the early 1900s. The contribution of genetic variants to susceptibility to MG has been actively studied, especially the contribution of single nucleotide polymorphisms (SNPs). A number of potential SNPs have been shown to be involved in MG pathogenesis, including variants of the major histocompatibility complex (MHC) genes [3] and non-MHC genes [4].

MicroRNAs (miRNAs) are small noncoding regulatory RNAs of 18–25 nucleotides that play important regulatory roles at the post-transcriptional level, which have been shown to be master gene regulators that control various cellular processes, including cell proliferation and differentiation, apoptosis, signal transduction, and immune responses [5]. Recent studies have revealed that aberrant miRNA expression might contribute to the initiation and progression of MG. For example, miRNA microarray analysis showed that 44 miRNAs were significantly dysregulated in MG [6]. The downregulation of miR-320a [7] and upregulation of miR-146a [8] in MG have also been validated and shown to be involved in pro-inflammatory cytokine expression and auto-antibody production. These findings have revealed that miRNAs have potential influences on MG pathogenesis.

Since the sequence complementarity and thermodynamics of the miRNA-mRNA binding are critical for their interactions, it is conceivable that miRNA-associated single nucleotide polymorphisms (miRSNPs) could affect miRNAs' regulatory function [9]. MiRSNPs have been classified into two kinds according to their locations, that is SNPs within miRNA genes and SNPs within miRNA target sites [10]. Recent studies have highlighted their significant roles in autoimmune diseases. SNPs within miRNAs genes could affect all states of miRNAs synthesis (pri-, pre-, and mature) and alter the biogenesis or function of miRNAs, and render them the primary causative genetic variants. rs57095329 in the miRNA-146a promoter has been shown to modulate the miRNA expression and has been confirmed to be linked to susceptibility to systemic lupus erythematosus (SLE) [11]. SNPs within miRNA target sites are much more common than variants within miRNAs [10]. This kind of miRSNPs may abolish, weaken or create a new miRNA target, disturbing the miRNA-mRNA interaction, and likely lead to a corresponding decrease or increase in protein translation [9]. rs3027898 (A>C) in the 3′UTR of *IRAK1* (interleukin-1 receptor-associated kinase), a target gene of miR-146a, has been shown to be involved in rheumatoid arthritis (RA) pathogenesis [12]. However, to date, few studies have elaborated the effects of miRSNPs in MG.

In this study, we systematically identified candidate functional miRSNPs and their potential mechanisms based on the current genetic findings for MG, which would further help to elucidate their potential roles in MG pathogenesis both in genetic variants and at the post-transcriptional regulation level.

## Materials and Methods

### Human myasthenia gravis risk gene data

We defined MG risk genes as genes with expression levels that were significantly different in the MG patient samples compared with the controls or genes that contain SNPs significantly associated with MG patients or subgroups. Information was obtained by querying the GAD (updated in August, 2011) [13] and Phenopedia (version 2.0) [14] databases and by manually reading literature published before April 1st, 2013, as revealed by searching the PubMed database using the terms “(myasthenia gravis [MeSH Terms]) AND English [Language]”. We thoroughly reviewed 8,896 items returned by our searches and selected MG risk genes that met the following criteria: (i) present in at least 5 MG samples (including peripheral blood samples and thymic tissue samples); (ii) the risk gene was detected using reliable biological experimental methods; (iii) a significantly different gene expression level (mRNA level or protein level) was identified using a t-test and a p-value cutoff of 0.05; and, (iv) the frequency of gene variants was significantly associated with MG prevalence (P<0.05). All of the risk genes were validated to be associated with MG by reliable biological experiments, and the p-values were partially adjusted for multiple testing.

### Pathway data

We obtained pathway data from the KEGG pathway database (updated in September 2013) [15] to identify MG risk pathways, and used the SubpathwayMiner package of R [16] to discover the pathways which each miRNA target gene assemblage was enriched in, namely the miRNA target pathways.

### miRNA data and miRNA target genes

Human miRNA information was acquired from miRBase (release 20) [17]. Human miRNA target data was obtained from ten miRNA target predicting tools, namely DIANA-microT (version 3.0) [18], mirSVR (August 2010 release) [19], PicTar5 (2013 July) [20], RNA22 (2013 July) [21], RNAhybrid (2013 July) [22], TargetScan (release 6.2) [23], PITA (version 6) [24], MirTarget2 (2013 July) [25], TargetMiner (2013 July) [26], and miRanda (August 2010 release) [27]. We narrowed down the target gene assemblages of each miRNA by extracting only miRNA-targets pairs that were predicted by at least four tools. Finally, 687 miRNAs, 16,110 miRNA target genes and 364,802 miRNA-target regulations were obtained.

### miRSNP data

We acquired the miRSNPs within miRNA target sites in six databases, namely Patrocles (2013 July) [28], miRNASNP (release 1.0) [29], miRdSNP (version 11.03) [30], PolymiRTS (version 3.0) [31], mirsnp (2013 July) [32], and dbSMR (2013 July) [33]. The miRSNPs within miRNAs were obtained from Patrocles (2013 July) [28], miRNA-SNiPer (version 3.9) [34], and miRNASNP (release 1.0) [29]; For both kinds of miRSNPs, we focused on the miRSNPs of interest being predicted by at least two databases, and all of these miRSNPs may potentially disrupt existing interactions between miRNA and mRNA.

### Pathway Enrichment Analysis

In order to identify the MG risk pathways which the MG risk genes were enriched in, we carried out KEGG pathway enrichment analysis by applying functional annotation tools in DAVID [35]. In the analysis, a significant level for the false discovery rate (FDR) set at less than 0.05 was defined as the cutoff. We also applied DAVID to obtain the Gene Ontology (GO) annotation [36] for the risk gene catalog. A GO term was considered significantly enriched if it displayed a FDR value less than 0.01.

### Cumulative hypergeometric distribution

We identified the crosstalk among MG risk pathways and the correlations between miRNAs and individual pathways using cumulative hypergeometric distribution [37], [38]. The formula was as follows:
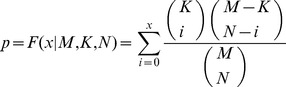



When analyzing pathway crosstalk, we supposed that the whole human genome had 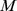 genes, and 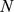 denoted the total number of genes in one risk pathway, 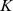 represented the total number of genes in another risk pathway, and 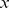 was the number of genes overlapping between these two pathways. The association between each MG risk pathway and all of the other pathways was analyzed. Similarly, for the identification of miRNA targeting pathways, 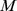 represented the number of genes in the whole human genome, 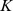 represented the number of genes in a given pathway, 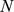 represented the number of target genes of a given miRNA, and 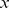 denoted the number of genes out of the total number of target genes that were involved in the pathway. We adjusted the p-value using the Benjamini and Hochberg false discovery rate to judge the statistical significance and we considered two pathways as significantly overlapped or a miRNA as targeting a pathway if the FDR was less than 0.01.

## Results

### Compiling human myasthenia gravis risk gene catalog

Through manual literature-mining, 89 MG risk genes were identified from 67 reliable studies (detailed information summarized in Table S1). These genes were validated in a total of 7,258 MG patients, ∼135,600 healthy controls and 140 patients with other neurological diseases. The genes were evaluated using typical experimental methods like PCR, ELISA, and TaqMan assays. The GO annotations of this MG risk gene set found they could be predominantly grouped into categories such as immune response, regulation of apoptosis, cytokine activity, and receptor binding (Figure S1), which was in concert with current knowledge of MG pathogenesis.

### Identifying human myasthenia gravis risk pathways

Based on the enrichment analysis of the MG risk gene catalog, we identified 18 MG risk pathways (Table 1). About 65% of the risk genes (58/89) were statistically associated with these pathways (FDR value<0.01), suggesting that we could use biological pathways to functionally characterize MG. Moreover, the majority of the identified pathways belonged to the categories of ‘human disease→immune system diseases’ and ‘Organismal systems→immune system’ in the KEGG database, highlighting the fundamental characteristics of autoimmune MG.

**Table 1:** Myasthenia gravis associated KEGG pathways.

KEGG pathway	FDR	Pathway maps
hsa05330:Allograft rejection	8.50E-12	Human diseases→immune diseases
hsa04060:Cytokine-cytokine receptor interaction	4.73E-11	Environmental information processing→signaling molecules and interaction
hsa04940:Type I diabetes mellitus	7.09E-11	Human diseases→endocrine and metabolic diseases
hsa05332:Graft-versus-host disease	1.01E-09	Human diseases→immune diseases
hsa05320:Autoimmune thyroid disease	1.27E-05	Human diseases→immune diseases
hsa04672:Intestinal immune network for IgA production	1.67E-04	Organismal systems→immune system
hsa04010:MAPK signaling pathway	1.83E-04	Environmental information processing→signal transduction
hsa05219:Bladder cancer	9.12E-04	Human diseases→cancers: specific types
hsa04630:Jak-STAT signaling pathway	0.0040	Environmental information processing→signal transduction
hsa04620:Toll-like receptor signaling pathway	0.0052	Organismal systems→immune system
hsa04650:Natural killer cell mediated cytotoxicity	0.0070	Organismal systems→immune system
hsa04660:T cell receptor signaling pathway	0.0092	Organismal systems→immune system
hsa04640:Hematopoietic cell lineage	0.0134	Organismal systems→immune system
hsa05200:Pathways in cancer	0.0143	Human diseases→cancers: overview
hsa05215:Prostate cancer	0.01733	Human diseases→cancers: specific types
hsa04722:Neurotrophin signaling pathway	0.02818	Organismal systems→nervous system
hsa05310:Asthma	0.03180	Human diseases→immune diseases
hsa05216:Thyroid cancer	0.03180	Human diseases→cancers: specific types

In addition, we explored the correlations among MG risk pathways by defining the overlap between every two pathways and constructed a pathway crosstalk network (Table S2, Figure S2A). The crosstalk network demonstrated that all of the biological pathways were significantly correlated with some of the rest. Three biological pathways involved in immune system response [hsa04660 (T cell receptor signaling pathway), hsa04650 (Natural killer cell mediated cytotoxicity), hsa04620 (Toll-like receptor signaling pathway)] widely correlated with 14 other pathways (Figure S2B). These results indicated that the MG risk pathways may be synergistic in the pathogenesis of MG, especially for the immune system response-related pathways.

### Constructing miRNA-mediated SNP switching pathway network

Because of the emerging role of miRNAs and their genetic variants in autoimmune disease susceptibility, we generated a pathway-based dissection of the roles of miRNAs and miRSNPs in MG. As a result, 198 significant miRNA-pathway links between 93 miRNAs and 9 MG risk pathways were identified (Table S3). To test the robustness of these miRNA-pathway links, we implemented a randomization test. For each miRNA-pathway link, we randomly selected 1000 gene sets with the same size of the pathway and calculated the overlap with miRNA targets. We found that all the empirical p-values (number of significant overlaps in random genes sets/1000) were less than 0.01, which demonstrates that the predicted miRNA-pathway links identified in our study were reliable. In addition, we also found that some of the associations between miRNAs and pathways have been reported and validated in previous studies: such as, miR-17 and hsa05219 (Bladder cancer pathway) [39], miR-20b and hsa05215 (Prostate cancer pathway) [40], and miR-21/16 and hsa04010 (MAPK signaling pathway) [41].

**Figure 1:**
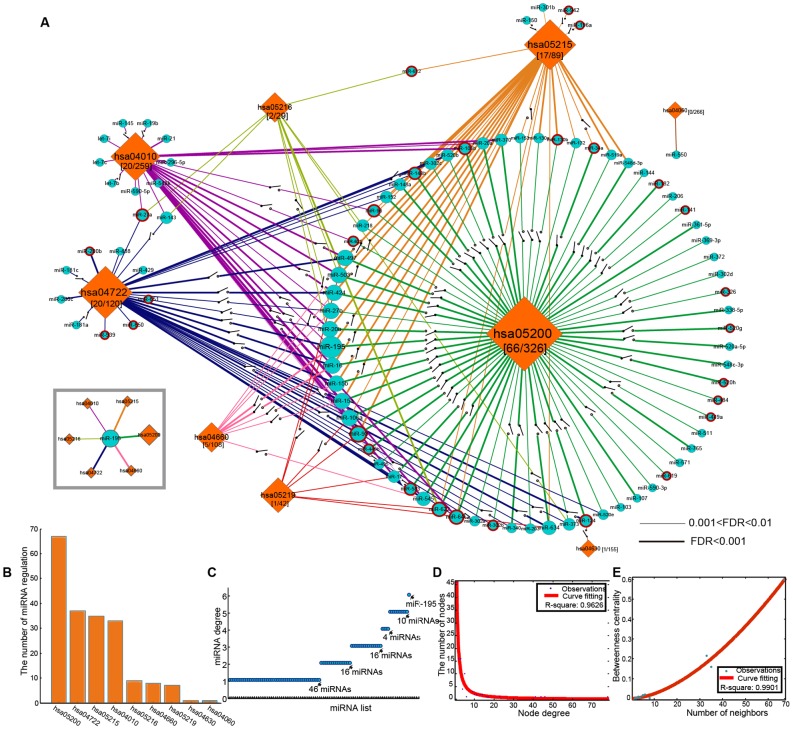
The MSSPN and its topological properties. (A) The MSSPN is composed of 9 risk pathways, 93 identified miRNAs and related miRSNPs. Orange rhombuses and blue circles represent pathways and miRNAs respectively, and their sizes correspond with their degrees. The SNPs within miRNA targets are represented with circuit ‘on-off’ symbol in physics locating on the lines between the miRNA and the pathway which contains the target gene; while the SNPs within miRNA are shown with the red circle around the miRNA. The number under a pathway name represents its SNP density. The lower left subfigure displays miR-195 and its regulations to six pathways. (B)the bar plot of pathways' degree distribution. (C)the scatter plot of miRNAs' degree distribution. Y-axis corresponds to miRNAs' degree; while x-axis to the 93 identified miRNAs (the concrete names are not shown). The total number of miRNAs with the same degree is remarked in the figure. (D) the node degree distribution of MSSPN and the curve fitting. (E) the betweenness centrality distribution of MSSPN and the curve fitting.

Furthermore, we searched for miRSNPs associated with these 93 miRNAs in seven important miRSNP databases, and screened for candidate functional miRSNPs, namely 91 significant SNPs within miRNA target sites which might affect the regulation of 46 miRNA involved in 8 MG risk pathways, and 42 significant miRSNPs within miRNAs which might be involved in the changes in base sequence or spatial structure of 29 miRNAs that could influence their ability to regulation the target pathway. A miRNA-mediated SNP switching pathway network (MSSPN) was also constructed to objectively reflect and elaborate the regulatory role of miRNAs and the potential influence of miRSNPs to MG at the pathway level (Figure 1A).

To identify the miRNAs or pathways with special significance, we calculated the degree distribution of the pathways (Figure 1B) and miRNAs (Figure 1C). Some pathways were found to link with the majority of the total 93 miRNAs. For example, hsa05200 (pathway in cancer) was regulated by 67 miRNAs (approximately 72% of the total miRNAs), while the four pathways with the highest degree of linkage [hsa05200 (pathway in cancer), hsa04722 (neurotrophin signaling pathway), hsa04010 (MAPK signaling pathway), hsa05215 (prostate cancer)] included about 99% (92 out of 93) of the total miRNAs, indicating that these four biological pathways were more likely to be regulated by the miRNAs. As for the miRNA degree distribution (Figure 1C), approximately one-sixth of the miRNAs regulated more than three risk pathways, especially for miR-195, which had interactions to six MG risk pathways (Figure 1A), suggesting they were probably more powerful genetic modulators in MG pathogenesis. MiR-195 has been widely investigated as an important inhibitory factor and therapeutic target in cancer, in consistent with its interactions with the three cancer-related pathways we identified. Just as we revealed that it could regulate has04722 (neurotrophin signaling pathway), miR-195 has also been suggested to play significant roles in the nervous system, including a contribution to the fine-tuning of brain-derived neurotrophic factor (BDNF) [42] and its close link to proliferation and differentiation of neural stem cells [43]. These findings support the idea that miR-195 might be a global genetic regulator in MG.

To assess which pathway contained the largest risk to be influenced by miRSNPs, we defined the SNP density of a pathway as the ratio of the number of genes which contained a miRSNP to the total number of genes in the pathway's gene set. As a result, hsa05200 (pathway in cancer) had the highest SNP density (66/326), indicating that it was more likely to be switched or influenced by miRSNPs.

Moreover, we also discussed the topological features of the MSSPN. The examination of the degree distribution (Figure 1D) revealed that the MSSPN was scale free. The betweenness centrality distribution (Figure 1E) showed that the node with more neighbors showed higher betweenness centrality, indicating that these nodes might play more important roles in the MSSPN. The pathway, hsa05200 (pathway in cancer), owned the highest betweenness centrality in our network, supporting its potential prominent role.

### Dissection of potential mechanisms of polymorphic ‘switch’ influencing miRNAs' regulation to myasthenia gravis risk pathway

#### Hsa05200 pathway in MG

**Figure 2:**
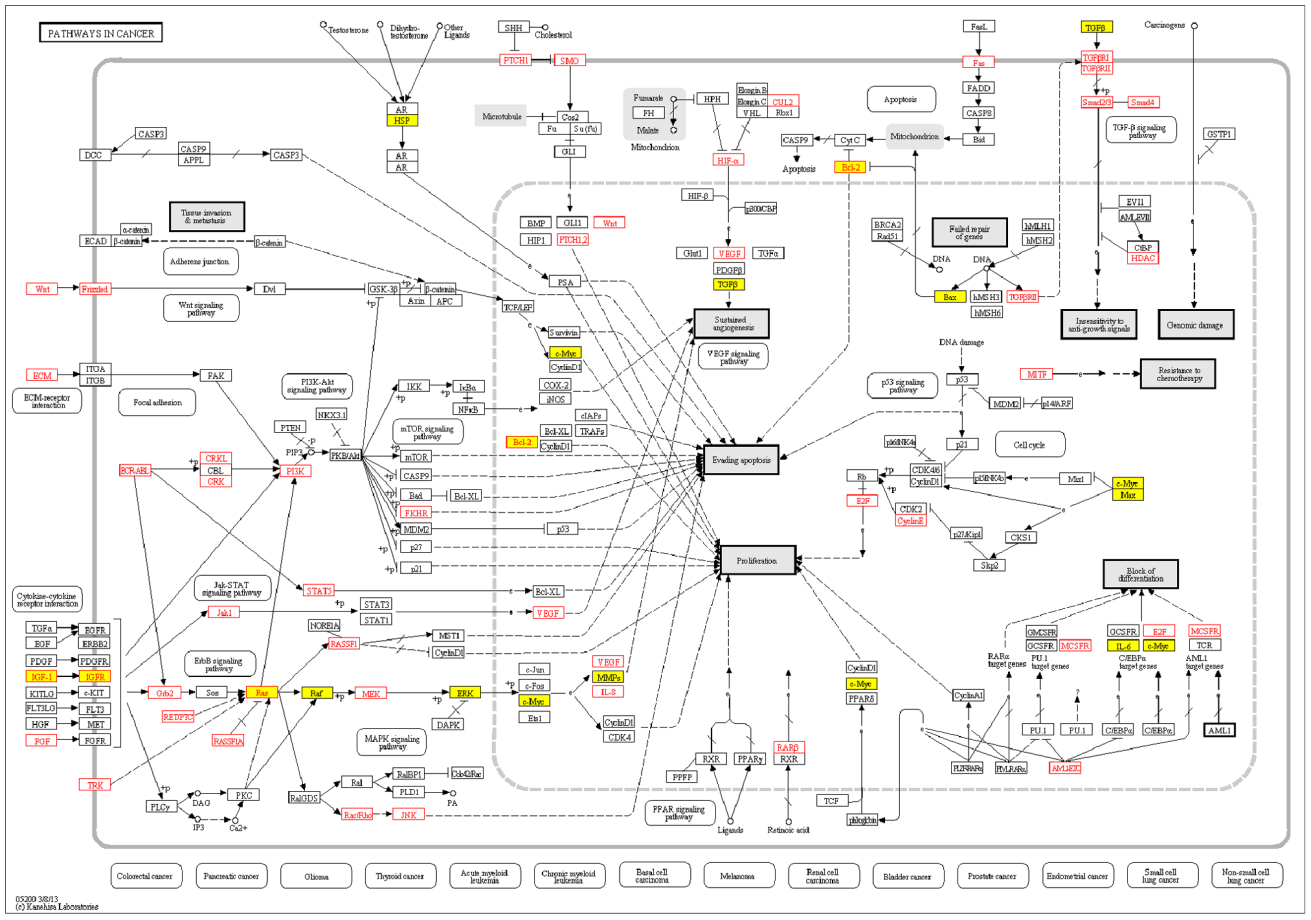
A depiction of the pathways in cancer in KEGG database. Proteins or complexes encoded by MG risk genes are indicated in yellow background, while those in red character represent that their encoding genes are the miRNA-target genes with miRSNPs in their 3′UTR regions. Four important genes (IGF1R, IGF1, RAS, and BCL2) are MG risk genes and meanwhile contain the miRSNPs, and are indicated in red character and yellow background.

Hsa05200 (pathway in cancer) has been revealed to be extremely significant in MG pathogenesis from the analysis above, as it was regulated by approximately 72% of the whole predicted miRNAs, showed its potential prominent role in the topological analysis of the MSSPN, and also contained the highest SNP density. Thus, we carried out an in-depth dissection on this particular pathway, and identified the location of those miRSNPs in the KEGG pathway map (Figure 2).

The miRSNPs were found to be located in genes that are members of many important subpathways in hsa05200 (pathway in cancer), such as the TGF-β signaling subpathway, the MAPK signaling subpathway, the cytokine-cytokine receptor interaction, and the RAS cascade response. Moreover, many of these miRSNPs are located in genes that encode first messenger signals and the cell surface receptors of these signaling subpathways, suggesting they might regulate the upstream activation of these subpathway. For example, the TGF-β signaling subpathway, one of the most important signaling pathways controlling a plethora of cellular responses, contains miRSNPs in the coding gene of both cell surface receptor for TGF-β (TGFβRI and TGFβRII) and their downstream second messengers. Importantly, several SNPs in TGF-β encoding genes have been validated to be significantly associated with ophthalmoplegic MG [44], highlighting the potential roles of this subpathway in MG.

#### IGF1R, IGF1, RAS, and BCL2 in MG

**Figure 3:**
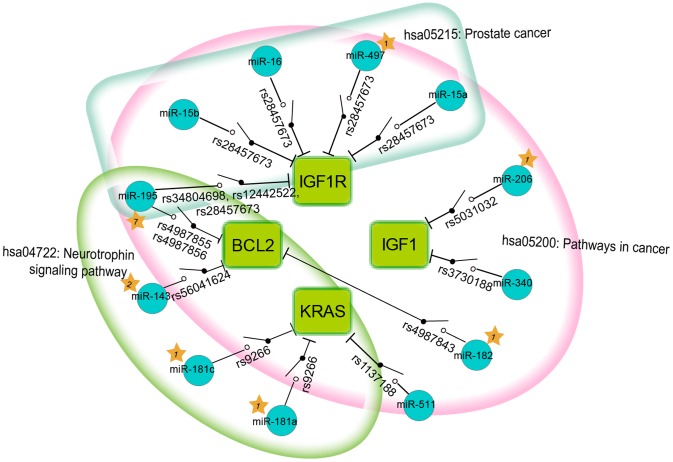
The schematic diagram of miRSNPs→gene→pathway effect of four MG high-risk genes. The green rectangles and blue circles represent the high-risk genes and their regulatory miRNAs respectively. The lines between them show the downregulation of miRNA to genes. The ‘on-off’ symbols sited on the line mean the miRSNPs within miRNA target sites. The peripheral large circles or rectangles denote the pathways that miRSNPs may influence through their effects on miRNAs' regulations to target genes. The orange stars with number inside beside miRNAs stand for the number of literatures validating the inhibition of miRNA to its target gene in Pubmed database.

From the dissection of hsa05200 (pathway in cancer), we revealed four high-risk genes (*IGF1R*, *IGF1*, *RAS*, and *BCL2*) to be MG risk genes and simultaneously showed they contained miRSNPs in their 3′UTR regions (Figure 2). The research focusing on miRSNPs in the high-risk genes would be more meaningful since these individual genes have already showed relatively definite roles in MG pathogenesis [45], [46], [47]. We characterized the potential biological mechanisms of miRSNPs→gene→pathway effect, which means that the miRSNPs might affect the MG pathway through regulating the function of a specific gene (Figure 3).

The four high-risk genes were found to be regulated by 12 miRNAs, while 7 of them have been experimentally verified to show that their downregulation targeted biologically relevant genes (Figure 3). Meanwhile, 11 miRSNPs were identified in the sequence of the high-risk genes, potentially influencing their expression and function, and which might further affect the status of hsa05200 (pathway in cancer) in MG. However, hsa05200 (pathway in cancer) might not be the only pathway affected, since hsa05215 (prostate cancer) might also be influenced by miR-15a, miR-15b, miR-16, miR-497, miR-195 and their miRSNPs via affecting *IGF1R*, and hsa04722 (neurotrophin signaling pathway) might be likewise influenced by miR-181a, miR-181c, miR-143, miR-195 and their miRSNPs via *KRAS* and *BCL2*. Overall, *IGF1R*, *IGF1*, *RAS*, and *BCL2* might be four high-risk genes in MG pathogenesis which could be regulated by many miRNAs and miRSNPs and influence several important MG risk pathways.

#### Influence of miRSNPs in MG

11 miRSNPs were identified that could potentially influence MG risk pathways via *IGF1R*, *IGF1*, *RAS*, and *BCL2* genes. In order to specify the influence of these miRSNPs, we dissected the features of these miRSNPs and proposed their potential mechanisms on the miRSNPs→gene→pathway effect.

**Figure 4:**
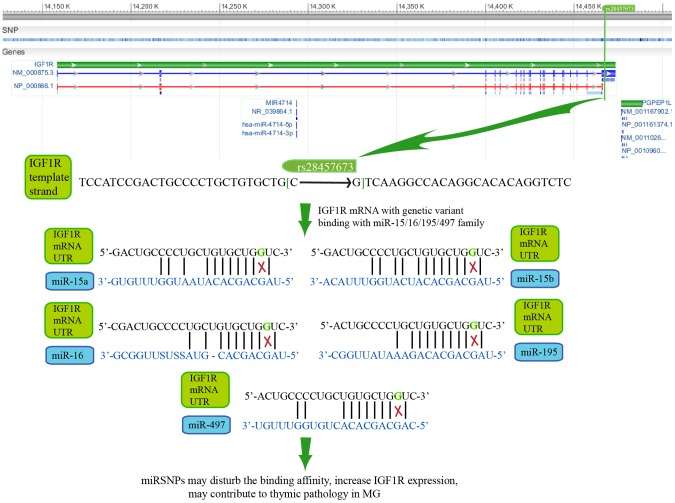
The potential mechanisms of rs28457673 influences IGF1R expression via miR-15/16/195/497 family.

The proteins encoded by *IGF1* and *IGF1R*, that is the insulin-like growth factor 1 and its high-affinity receptor, have been widely investigated. Overall, amplified Igf1/Igf1R signaling can promote the proliferation and differentiation of lymphocytes, especially in thymus [48]. Previous studies had found an increase in the distribution of Igf1R and Igf1 positive epithelial cells in the pathological thymus of MG patients [45]. Our analysis and literature mining revealed that both *IGF1* and *IGF1R* contained miRSNPs which might influence their functions. rs28457673 is a genetic variant C>G in the 3′UTR region of *IGF1R* that may affect the affinity of interactions between *IGF1R* mRNA and several miRNAs, such as miR-497, miR-15a, miR-15b, miR-16, and miR-195 (Figure 4). These miRNAs belong to one miRNA family (the miR-15/16/195/424/497 family), whose members share the same 3′UTR binding seed sequence and have been reported to cooperatively regulate many targets [49]. MiR-497 has been validated to inhibit *IGF1R* expression via targeting *IGF1R* mRNA in human colorectal cancer [48], which strengthens our speculation that the other 4 miRNAs inhibit *IGF1R*. Moreover, one recent miRNA microarray analysis has shown a decreased level of miR-15a, miR-15b and miR-16 in MG patients [6], which might be the cause of the increased *IGF1R* in the MG patients' thymus. The accumulated analysis supported the potential mechanism suggesting rs28457673 (miR-15/16/195/424/497 family)→*IGF1R*→hsa05200 (pathway in cancer)/hsa05215 (prostate cancer) might play important roles in MG pathogenesis.

Another important high-risk gene in cancer, *BCL2*, encoding the apoptosis regulator protein, Bcl-2, has also been reported to be expressed at increased levels in MG thymus in several studies, including one which showed that the expression levels of the Bcl2 family genes correlated with MG severity [46]. The fine-tuning of miRNA to *BCL2* has been established, and miR-195 is the best characterized of the miRNA [50]. rs4987856, a C>T variant in the miR-195 binding site, has been demonstrated to impact the interaction of miR-195 and *BCL2* as determined by secondary structure prediction and binding energy determination in a recent study [51]. It has strengthened the potential mechanism of the rs4987856 (miR-195)→*BCL2*→MG risk pathways effect.

MiRSNP rs9266, a T>C variant in the high-risk gene KRAS, potentially disturbs KRAS mRNA inhibition by miR-181a and miR-181c and therefore could influence the significant role that K-ras plays in hsa04722 (neurotrophin signaling pathway). The inhibition of the miR-181 family in thymocytes could decrease TCR sensitivity and impair positive and negative T cell selection in the thymus, which are essential for the immunotolerance process and autoimmune disease [52], [53]. Moreover, the expression of miR-181c has been demonstrated to be significantly down-regulated in a recent microarray study of an experimental autoimmune MG rat model [54]. miR-181c downregulation might influence the pathogenesis of MG by causing the over-expression of KRAS [47] and by disturbing immunotolerance in the thymus.

## Discussion

The dissection of the ‘switch’ of miRSNPs on miRNAs' regulation on relevant pathways would help to elucidate their potential roles in MG pathogenesis both as genetic variants and at the post-transcriptional regulation level. In this study, we have for the first time, systematically identified candidate functional miRSNPs and their potential mechanisms based on the current genetic findings of MG. Through manually compiling the MG risk gene catalog, we enriched MG risk pathways, and then identified miRNAs targeting MG risk pathways. Furthermore, we revealed the candidate functional miRSNPs ‘switches’ in the miRNAs that regulate MG risk pathways by searching and screening reliable miRSNPs database information, and constructed the MSSPN. In addition, we carried out an in-depth dissection of the correlation with hsa05200 (pathway in cancer), elaborated the significance of four high-risk genes, and proposed the potential mechanisms of particular miRSNPs as ‘switches’ in miRNAs regulation of the MG risk pathways.

The 18 MG risk pathways we identified provides an overview of MG pathogenesis and reflects the macroscopic effect of dysfunctions in MG risk genes and gene modulators. They may also reveal the latent relationship between MG and other disorders, for example, ‘hsa05330 (allograft rejection)’ was revealed to have the most significant relationship with MG at a biological pathway level, which was in consistent with several case-reports of MG arising after allogeneic bone marrow transplantation or as a manifestation of chronic graft-versus-host-disease [55]. Another potential advantage of our pathway analysis approach is that it may provide insights into identification of disease subtypes. MG is heterogeneous in its clinical manifestations. Pathway-based genetic analyses may help identify different, and even unrelated, biological mechanisms as responsible for similar disease pathogenesis. The implications of such potential discovery are broad, because it might lead to targeted therapeutics and individual treatment. Here, we found a close link between MG and cancer in the pathway view (Table 1), supporting the notion of paraneoplastic MG, meaning the pathogenesis of MG is highly related with neoplasms, as another important subtype of MG [56].

MiRNAs, which are important post-transcriptional genetic regulators of gene expression, have the power to regulate diverse biological pathways simultaneously because each miRNA is capable of targeting a large number of genes. We identified 93 miRNAs that have the potential to regulate MG risk pathways and that were found to be more significant than those miRNAs targeting individual MG risk genes. Compared with one recent miRNA microarray analysis [6], which had found 44 miRNAs to be significantly dysregulated in MG, 16 miRNAs were simultaneously predicted in our potential miRNA profile, including 11 down-regulated miRNAs and 5 up-regulated miRNAs. A microarray-based study had previously validated that miR-145, one of the functionally associated miRNAs we predicted, was one of the most significantly down-regulated miRNAs in experimental autoimmune MG rat models and its down-regulation could promote pathogenic Th17 cell responses in MG [54].

According to the topological properties of MSSPN and detailed mechanism dissection, we revealed the potentially significant roles of hsa05200 (pathway in cancer), four high-risk genes (*IGF1R*, *IGF1*, *RAS*, and *BCL2*), and associated miRSNPs in MG. Through mechanism-seeking of the miRSNPs in the MG high-risk genes, we identified the three most strongly suggested potential mechanisms of miRSNP→gene→pathway effect, as follows: (i) rs28457673(miR-15/16/195/424/497 family)→*IGF1R*→hsa05200 (pathway in cancer)/hsa05215 (prostate cancer); (ii) rs4987856 (miR-195)→*BCL*→hsa05200 (pathway in cancer)/hsa04722 (neurotrophin signaling pathway); (iii) rs9266 (miR-181a/181c)→*KRAS*→hsa04722 (neurotrophin signaling pathway); however, these potential mechanisms should be interpreted with caution since there is not definite experimental evidence for many steps, Nonetheless, our approach has demonstrated the potential value of in-depth studies using rich database resources that exist together with a pathway analysis approach supplemented by literature mining. Our analysis relies on the annotation of genes and the use of databases. Along with the accumulation of data, our research could serve as an important complement to future experimental studies of miRNA and miRSNPs in MG, especially given the lack of exploration in this field to date. In addition, previous studies have found that MG has several subtypes, and the pathogenesis and genetic predisposition might differ in these MG subtypes [57], [58]. We can incorporate more sufficient subtype information of MG in future studies, to make our results increasingly useful.

In summary, we created a risk gene catalog of MG, obtained the MG risk pathways, identified functional-associated miRNAs and miRSNPs regulating MG risk pathways, constructed the MSSPN, and finally revealed the significant roles of hsa05200 (pathway in cancer), four high-risk genes and related miRSNPs. Our research has demonstrate one method to reveal the functional role of genetic modulators in MG pathogenesis through a systemic level. Further in vitro and in vivo experiments would be necessary to confirm the potential roles of miRNAs and miRSNPs in MG pathogenesis.

## Supporting Information

Figure S1 **Gene Ontology annotations of MG risk gene catalog (displaying the first 30 items and their significances).**
(PDF)

Figure S2 **The crosstalk among myasthenia gravis risk pathways.** (A) the pathway-pathway network to demonstrate the significantly overlapped pathways among MG risk pathways. The orange rhombuses stand for each risk pathway, and the lines between two rhombuses stand for the significant correlation between two pathways. The lines in light grey represent the FDR value was less than 0.01, but more than 0.001, while the lines in dark grey denote the FDR value was less than 0.001, meaning the two pathways are more significantly overlapped. (B) the bar plot of pathway's degree distributions in the network. The average degree of pathways is 10.444, while hsa04660, hsa04650 and hsa04620 share the highest degree (14).
(PDF)

Table S1 **The catalog of MG risk genes.**
(DOC)

Table S2 **The detail information of overlapped myasthenia gravis risk pathways.**
(XLS)

Table S3 **The details of microRNA regulation to myasthenia gravis risk pathways.**
(XLS)
