# Supplementary figures and images for: Identifying a Polymorphic ‘Switch’ That Influences miRNAs' Regulation of a Myasthenia Gravis Risk Pathway
Source: PLoS One. 2014 Aug 12;9(8):e104827. doi: 10.1371/journal.pone.0104827 (PMC4130595; doi:10.1371/journal.pone.0104827)

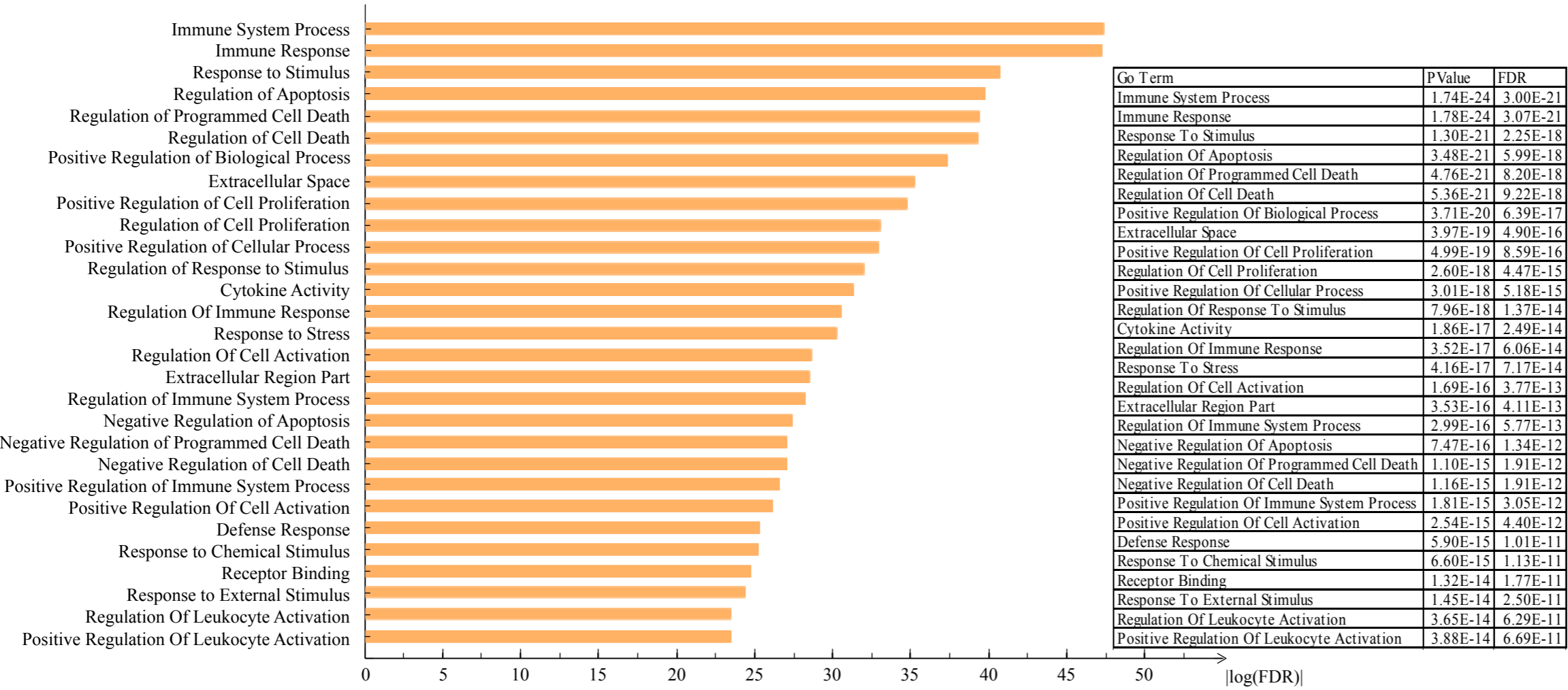

Supplement: Figure S1 — Gene Ontology annotations of MG risk gene catalog (displaying the first 30 items and their significances). (PDF) [file pone.0104827.s001.pdf]

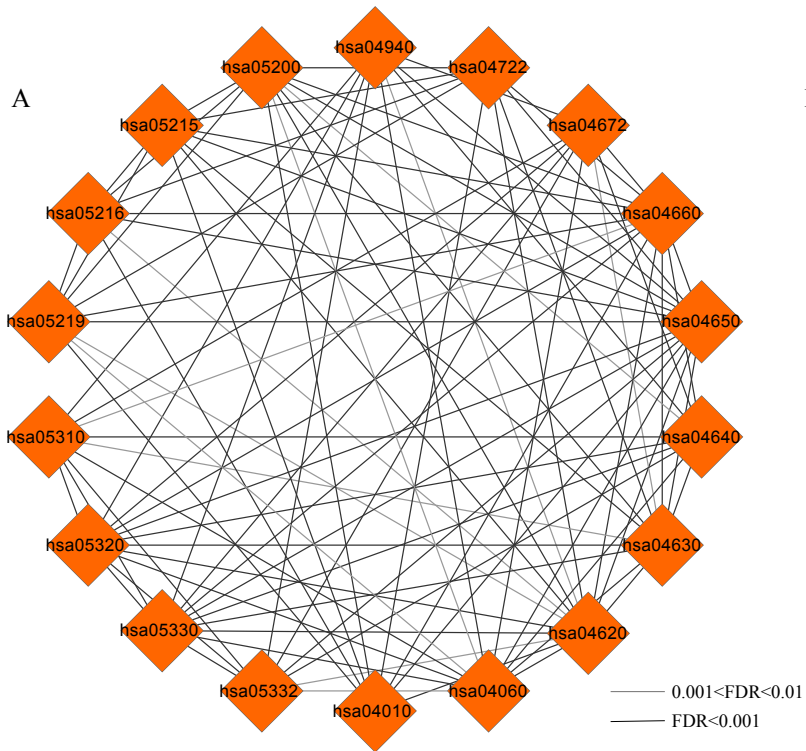

B

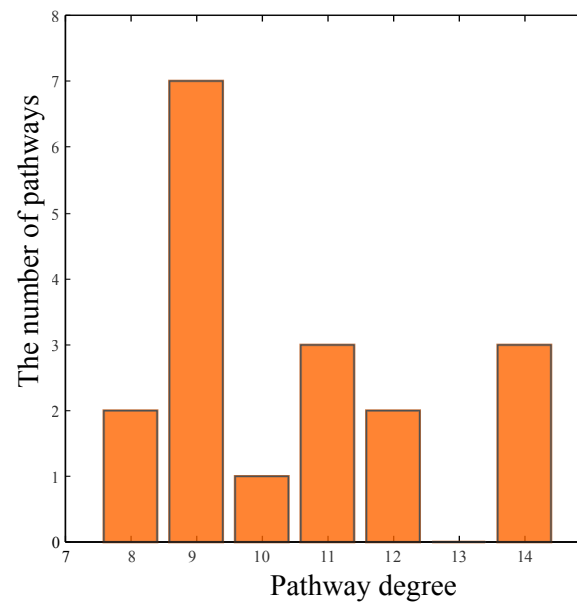

Supplement: Figure S2 — The crosstalk among myasthenia gravis risk pathways. (A) the pathway-pathway network to demonstrate the significantly overlapped pathways among MG risk pathways. The orange rhombuses stand for each risk pathway, and the lines between two rhombuses stand for the significant correlation between two pathways. The lines in light grey represent the FDR value was less than 0.01, but more than 0.001, while the lines in dark grey denote the FDR value was less than 0.001, meaning the two pathways are more significantly overlapped. (B) the bar plot of pathway's degree distributions in the network. The average degree of pathways is 10.444, while hsa04660, hsa04650 and hsa04620 share the highest degree (14). (PDF) [file pone.0104827.s002.pdf]
