# Supplementary material for: Identifying a Polymorphic ‘Switch’ That Influences miRNAs' Regulation of a Myasthenia Gravis Risk Pathway
Source: PLoS One. 2014 Aug 12;9(8):e104827. doi: 10.1371/journal.pone.0104827 (PMC4130595; doi:10.1371/journal.pone.0104827)
Supplement: Table S1 — The catalog of MG risk genes. (DOC) [file pone.0104827.s003.doc]

**Supplementary table S1. The catalog of MG** risk genes.

| gene | Gene ID | Description | results | Expression | Detection method | Samples | Reference | Year |
| --- | --- | --- | --- | --- | --- | --- | --- | --- |
| ERBB4 | 2066 | This gene is a member of the Tyr protein kinase family and the epidermal growth factor receptor subfamily. It encodes a single-pass type I membrane protein with multiple cysteine rich domains, a transmembrane domain, a tyrosine kinase domain, a phosphotidylinositol-3 kinase binding site and a PDZ domain binding motif. The protein binds to and is activated by neuregulins and other factors and induces a variety of cellular responses including mitogenesis.and differentiation。Mutations in this gene have been associated with cancer. | Results show reduced mRNA expression levels of  ErbB4 in thymus tissue of iMG patients compared to AChR-MG (*P*＜0.0001) and non-MG patients(*P*= 0.0175). | down-regulation | Microarray experiments | 13 MG patients/14 HC | 21168922 | 2011[^1^](#_ENREF_1) |
| MYC | 4609 | The protein encoded by this gene is a multifunctional, nuclear phosphoprotein that plays a role in cell cycle progression, apoptosis and cellular transformation. It functions as a transcription factor that regulates transcription of specific target genes. Mutations, overexpression, rearrangement and translocation of this gene have been associated with a variety of hematopoietic tumors, leukemias and lymphomas, including Burkitt lymphoma. | The mRNA levels of c-myc were markedly reduced in MG thymusescompared to control (*P=*0.0027). | down-regulation | RT-PCR | 8 MG patients/6 HC | 11282171 | 2001[^2^](#_ENREF_2) |
| MAX | 4149 | The protein encoded by this gene is a member of the basic helix-loop-helix leucine zipper (bHLHZ) family of transcription factors. It is able to form homodimers and heterodimers with other family members, which include Mad, Mxi1 and Myc. | The mRNA levels of max were markedly reduced in MG thymuses compared with that in control thymuses (*P=*0.0002). | down-regulation | RT-PCR | 8 MG patients/6 HC | 11282171 | 2001[^2^](#_ENREF_2) |
| ACHE | 43 | Acetylcholinesterase hydrolyzes the neurotransmitter, acetylcholine at neuromuscular junctions and brain cholinergic synapses, and thus terminates signal transmission. It is encoded by the single ACHE gene, and the structural diversity in the gene products arises from alternative mRNA splicing, and post-translational associations of catalytic and structural subunits. | The AChE-S variant mRNA was decreased in thymoma patients compared with hyperplasia (*P*≤0.02) and healthy adults (*P*≤0.012). AChE-R mRNA was increased in thymoma patients with MG compared with the common AChE (*P=*0.06) and AChE-S (*P* ≤0.02). | AChE-S: down-regulation AChE-R: up-regulation | Real-time RT-PCR | 9 MG patients/4 HC | 17272501 | 2007[^3^](#_ENREF_3) |
| CCL21 | 6366 | This gene is one of several CC cytokine genes clustered on the p-arm of chromosome 9. Cytokines are a family of secreted proteins involved in immunoregulatory and inflammatory processes. Protein encoded by this gene inhibits hemopoiesis and stimulates chemotaxis. This protein is chemotactic in vitro for thymocytes and activated T cells, It is a high affinity functional ligand for chemokine receptor 7 (CCR7) that is expressed on T and B lymphocytes. | High overexpression of CCL21 in all MG patients with high thymic hyperplasia thymuses (MH) compared with babies(BB) (*P*=0.0019), non-MG adults (*P*=0.0002) (AD), MG patients with low thymic hyperplasia (ML) (*P*=0.0005) | up-regulation | ELISA | 21MH/12BB、12AD、12ML | 19847900 | 2009[^4^](#_ENREF_4) |
| CCL19 | 6363 | This gene is one of several CC cytokine genes clustered on the p-arm of chromosome 9. Cytokines are a family of secreted proteins involved in immunoregulatory and inflammatory processes. The cytokine encoded by this gene may play a role in normal lymphocyte recirculation and homing. It also plays an important role in trafficking of T cells in thymus, and in T cell and B cell migration to secondary lymphoid organs. | An increased expression of CCL19 was also observed in MG patients with high thymic hyperplasia thymuses (MH) as compared with thymuses from non-MG adults (*P*=0.028) and MG patients with low thymic hyperplasia (ML) (*P*=0.002) | up-regulation | ELISA | 21MH/12AD、12ML | 19847900 | 2009[^4^](#_ENREF_4) |
| LGALS8 | 3964 | This gene encodes a member of the galectin family. The galectins have been implicated in many essential functions including development, differentiation, cell-cell adhesion, cell-matrix interaction, growth regulation, apoptosis, and RNA splicing. | A significant association of anti-AChR antibody-positive MG with the rs237713 polymorphism (*P*= 0.008). | Positive association | TaqMan Allelic Discrimination Assay | 149 MG patients/365 HC | 22683700 | 2012[^5^](#_ENREF_5) |
| MAPK1 | 5594 | The protein encoded by this gene is a member of the MAP kinase family. MAP kinases, also known as extracellular signal-regulated kinases (ERKs), act as an integration point for multiple biochemical signals, and are involved in a wide variety of cellular processes such as proliferation, differentiation, transcription regulation and development | MAPK1 genes are basally overexpressed in MG-thymic epithelial cells. | up-regulation | macroarray | 8 MG patients/9 HC | 16272363 | 2005[^6^](#_ENREF_6) |
| MAP3K1 | 4214 | The protein encoded by this gene is a serine/threonine kinase and is part of some signal transduction cascades, including the ERK and JNK kinase pathways as well as the NF-kappa-B pathway | MAP3K1 genes are basally overexpressed in MG-thymic epithelial cells | up-regulation | macroarray | 8 MG patients/9 HC | 16272363 | 2005[^6^](#_ENREF_6) |
| MAP3K3 | 4215 | This protein directly regulates the stress-activated protein kinase (SAPK) and extracellular signal-regulated protein kinase (ERK) pathways by activating SEK and MEK1/2 respectively. In cotransfection assays, it enhanced transcription from a nuclear factor kappa-B (NFKB)-dependent reporter gene, consistent with a role in the SAPK pathway | MAP3K3 genes are basally overexpressed in MG-thymic epithelial cells | up-regulation | macroarray | 8 MG patients/9 HC | 16272363 | 2005[^6^](#_ENREF_6) |
| MAP3K4 | 4216 | This gene encodes a MAPKKK, the MEKK4 protein, also called MTK1. This protein contains a protein kinase catalytic domain at the C terminus. The N-terminal nonkinase domain may contain a regulatory domain. Expression of MEKK4 in mammalian cells activated the CSBP2 and JNK MAPK pathways, MEKK4 is a major mediator of environmental stresses that activate the CSBP2 MAPK pathway, and a minor mediator of the JNK pathway. | MAP3K4 genes are basally overexpressed in MG-thymic epithelial cells | up-regulation | macroarray | 8 MG patients/9 HC | 16272363 | 2005[^6^](#_ENREF_6) |
| MAP3K11 | 4296 | The protein encoded by this gene is a member of the serine/threonine kinase family. This kinase preferentially activates MAPK8/JNK kinase, and functions as a positive regulator of JNK signaling pathway. This kinase can directly phosphorylate, and activates IkappaB kinase alpha and beta, and is found to be involved in the transcription activity of NF-kappaB mediated by Rho family GTPases and CDC42 | MAP3K11 genes are basally overexpressed in MG-thymic epithelial cells | up-regulation | macroarray | 8 MG patients/9 HC | 16272363 | 2005[^6^](#_ENREF_6) |
| BRAF | 673 | This gene encodes a protein belonging to the raf/mil family of serine/threonine protein kinases. This protein plays a role in regulating the MAP kinase/ERKs signaling pathway, which affects cell division, differentiation, and secretion. | BRAF genes are basally overexpressed in MG-thymic epithelial cells | up-regulation | macroarray | 8 MG patients/9 HC | 16272363 | 2005[^6^](#_ENREF_6) |
| NRAS | 4893 | This is an N-ras oncogene encoding a membrane protein that shuttles between the Golgi apparatus and the plasma membrane. The encoded protein, which has intrinsic GTPase activity, is activated by a guanine nucleotide-exchange factor and inactivated by a GTPase activating protein. Mutations in this gene have been associated with somatic rectal cancer, follicular thyroid cancer, autoimmune lymphoproliferative syndrome, Noonan syndrome, and juvenile myelomonocytic leukemia | NRAS genes are basally overexpressed in MG-thymic epithelial cells | up-regulation | macroarray | 8 MG patients/9 HC | 16272363 | 2005[^6^](#_ENREF_6) |
| HRAS | 3265 | This gene belongs to the Ras oncogene family, whose members are related to the transforming genes of mammalian sarcoma retroviruses. The products encoded by these genes function in signal transduction pathways. These proteins can bind GTP and GDP, and they have intrinsic GTPase activity. | HRAS genes are basally overexpressed in MG-thymic epithelial cells | up-regulation | macroarray | 8 MG patients/9 HC | 16272363 | 2005[^6^](#_ENREF_6) |
| KRAS | 3845 | This gene, a Kirsten ras oncogene homolog from the mammalian ras gene family, encodes a protein that is a member of the small GTPase superfamily. The transforming protein that results is implicated in various malignancies, including lung adenocarcinoma, mucinous adenoma, ductal carcinoma of the pancreas and colorectal carcinoma. | KRAS genes are basally overexpressed in MG-thymic epithelial cells | up-regulation | macroarray | 8 MG patients/9 HC | 16272363 | 2005[^6^](#_ENREF_6) |
| DUSP1 | 1843 | The expression of DUSP1 gene is induced in human skin fibroblasts by oxidative/heat stress and growth factors. The bacterially expressed and purified DUSP1 protein has intrinsic phosphatase activity, and specifically inactivates mitogen-activated protein (MAP) kinase in vitro by the concomitant dephosphorylation of both its phosphothreonine and phosphotyrosine residues. Furthermore, it suppresses the activation of MAP kinase by oncogenic ras in extracts of Xenopus oocytes. | DUSP1 genes are basally overexpressed in MG-thymic epithelial cells | up-regulation | macroarray | 8 MG patients/9 HC | 16272363 | 2005[^6^](#_ENREF_6) |
| IL6 | 3569 | This gene encodes a cytokine that functions in inflammation and the maturation of B cells. The protein is primarily produced at sites of acute and chronic inflammation, where it is secreted into the serum and induces a transcriptional inflammatory response through interleukin 6 receptor, alpha. The functioning of this gene is implicated in a wide variety of inflammation-associated disease states, including suspectibility to diabetes mellitus and systemic juvenile rheumatoid arthritis | IL-6 levels were abnormally augmented in MG- thymic epithelial cells than in control thymuses of age-matched donors following stimulation by adhesive stimuli (*P*＜0.05) | up-regulation | ELISA | 8 MG patients/9 HC | 16272363 | 2005[^6^](#_ENREF_6) |
| CCL5 | 6352 | This gene is one of several CC cytokine genes clustered on the q-arm of chromosome 17. Cytokines are a family of secreted proteins involved in immunoregulatory and inflammatory processes. The cytokine encoded by this gene functions as a chemoattractant for blood monocytes, memory T helper cells and eosinophils. This cytokine is one of the major HIV-suppressive factors produced by CD8+ cells. | RANTES levels were abnormally augmented in MG-thymic epithelial cells than in control thymuses of age-matched donors following stimulation by adhesive stimuli( *P*＜0.05) | up-regulation | ELISA | 8 MG patients/9 HC | 16272363 | 2005[^6^](#_ENREF_6) |
| GZMB | 3002 | They are thought to protect their host by lysing cells bearing on their surface 'nonself' antigens, usually peptides or proteins resulting from infection by intracellular pathogens. The protein encoded by this gene is crucial for the rapid induction of target cell apoptosis by CTL in cell-mediated immune response. | GrB efficiently and specifically cleaves subunits of AChR, especially the epsilon subunit. GrB is present in thymus glands from myasthenia patients, but is absent in control thymuses. | up-regulation | immunoblotting | 5 MG patients/5 HC | 18675462 | 2008[^7^](#_ENREF_7) |
| HLA-A | 3105 | HLA-A belongs to the HLA class I heavy chain paralogues. This class I molecule is a heterodimer consisting of a heavy chain and a light chain (beta-2 microglobulin). The heavy chain is anchored in the membrane. Class I molecules play a central role in the immune system by presenting peptides derived from the endoplasmic reticulum lumen. They are expressed in nearly all cells. The heavy chain is approximately 45 kDa and its gene contains 8 exons. Exon 1 encodes the leader peptide, exons 2 and 3 encode the alpha1 and alpha2 domains, which both bind the peptide, exon 4 encodes the alpha3 domain, exon 5 encodes the transmembrane region, and exons 6 and 7 encode the cytoplasmic tail. Polymorphisms within exon 2 and exon 3 are responsible for the peptide binding specificity of each class one molecule. | The present study clearly showed associations with -A1(*P=*0.010) and -A2(*P=*0.030) in MG. In patients with early-onset MG, associations with -A2 were stronger (adjusted by Yates’ correction) . | Positive association | complement-dependent microcytotoxicity assays | 66 MG patients/188 HC | 15301866 | 2004[^8^](#_ENREF_8) |
|  |  |  | Patients with myasthenia gravis (MG) and controls has revealed positive associations of HLA classI A*31(*P*＜0.05) .and A*31 indicated increased frequencies among patients with thymic hyperplasia versus patients without hyperplasia or controls(*P*＜0.05) (p-values were corrected multiplying by the number of comparisons). | Positive association | sequence-specific oligonucleotide probes | 49 MG patients/160 HC | 14700596 | 2004[^9^](#_ENREF_9) |
|  |  |  | The largest effect was a protection associated with HLA-A* 02 in MG patients with a B2 type thymoma (*P*=0.00041). The frequency of HLA- A*25 was also increased in the whole group of patients (*P*=0.0041). | Negative associations or Positive association | typing kit from Dynal Biotech | 78 MG patiens/122953 HC | 19278738 | 2009[^10^](#_ENREF_10) |
|  |  |  | HLA-A*23 were more frequent in MG cases than controls(*P*=0.04) | Positive association | PCR-SSP | 109 MG patients/383 HC | 19490212 | 2009[^11^](#_ENREF_11) |
| HLA-B | 3106 | HLA-B belongs to the HLA class I heavy chain paralogues. This class I molecule is a heterodimer consisting of a heavy chain and a light chain (beta-2 microglobulin). The heavy chain is anchored in the membrane. Class I molecules play a central role in the immune system by presenting peptides derived from the endoplasmic reticulum lumen. They are expressed in nearly all cells. The heavy chain is approximately 45 kDa and its gene contains 8 exons. Exon 1 encodes the leader peptide, exon 2 and 3 encode the alpha1 and alpha2 domains, which both bind the peptide, exon 4 encodes the alpha3 domain, exon 5 encodes the transmembrane region and exons 6 and 7 encode the cytoplasmic tail. Polymorphisms within exon 2 and exon 3 are responsible for the peptide binding specificity of each class one molecule | The present study clearly showed associations with -B8 (*P=*0.000) in MG. In patients with early-onset MG, associations with -B8 were stronger(adjusted by Yates’ correction). | Positive association | complement-dependent microcytotoxicity  assays | 66 MG patients /188 HC | 15301866 | 2004[^8^](#_ENREF_8) |
|  |  |  | Patients with myasthenia gravis (MG) and controls has revealed positive associations of HLA classI B*08, B*39, B*40 (*P*＜0.05).And B*08 indicated increased frequencies among patients with thymic hyperplasia versus patients without hyperplasia or controls(*P*＜0.05) (p-values were corrected multiplying by the number of comparisons). | Positive association | sequence-specific oligonucleotide probes | 49 MG patients /160 HC | 14700596 | 2004[^9^](#_ENREF_9) |
|  |  |  | HLA-B*08(*P*=0.00001), *18 (*P*=0.02)and*51(*P*= 0.0001) were all increased in frequency in the MG group. HLA-B*50 was significantly reduced in the MG group compared with controls | Positive association or negative association | PCR-SSP | 109 MG patients /383 HC | 19490212 | 2009[^11^](#_ENREF_11) |
| HLA-C | 3107 | HLA-C belongs to the HLA class I heavy chain paralogues. This class I molecule is a heterodimer consisting of a heavy chain and a light chain (beta-2 microglobulin). The heavy chain is anchored in the membrane. Class I molecules play a central role in the immune system by presenting peptides derived from endoplasmic reticulum lumen. They are expressed in nearly all cells. The heavy chain is approximately 45 kDa and its gene contains 8 exons. Exon one encodes the leader peptide, exons 2 and 3 encode the alpha1 and alpha2 domain, which both bind the peptide, exon 4 encodes the alpha3 domain, exon 5 encodes the transmembrane region, and exons 6 and 7 encode the cytoplasmic tail. Polymorphisms within exon 2 and exon 3 are responsible for the peptide binding specificity of each class one molecule | Patients with MG and controls has revealed positive associations of HLA classI C*15(*P*＜0.05), C*17(*P*＜0.05). And C*17 indicated increased frequencies among patients with thymic hyperplasia versus patients without hyperplasia or controls(*P*＜0.05) (p-values were corrected multiplying by the number of comparisons). | Positive association | sequence-specific oligonucleotide probes | 49 MG patients /160 HC | 14700596 | 2004[^9^](#_ENREF_9) |
| HLA-DQA1 | 3117 | HLA-DQA1 belongs to the HLA class II alpha chain paralogues. The class II molecule is a heterodimer consisting of an alpha (DQA) and a beta chain (DQB), both anchored in the membrane. It plays a central role in the immune system by presenting peptides derived from extracellular proteins. Class II molecules are expressed in antigen presenting cells (APC: B Lymphocytes, dendritic cells, macrophages). The alpha chain is approximately 33-35 kDa. It is encoded by 5 exons; exon 1 encodes the leader peptide, exons 2 and 3 encode the two extracellular domains, and exon 4 encodes the transmembrane domain and the cytoplasmic tail. Within the DQ molecule both the alpha chain and the beta chain contain the polymorphisms specifying the peptide binding specificities, resulting in up to four different molecules. | DQA1*05 indicated increased frequencies among patients with thymic hyperplasia versus patients without hyperplasia or controls(*P*＜0.05) (p-values were corrected multiplying by the number of comparisons). | Positive association | sequence-specific oligonucleotide probes | 49 MG patients /160 HC | 14700596 | 2004[^9^](#_ENREF_9) |
|  |  |  | DQA1*0401(*P=*0.008) had a highly significant association in all MG patients compared to the controls. DQA1*0103 (*P* = 0.000)was less frequent in patients compared to the controls, confirming the protective effect of these alleles for MG. Patients with thymomatous MG were positively associated with DQA1 *0401(*P=*0.011)as compared to MG patients without thymoma. | Positive association or negative associations | PCR-SSP | 84 MG patients /293 HC | 21917268 | 2012[^12^](#_ENREF_12) |
| HLA-DQB1 | 3119 | HLA-DQB1 belongs to the HLA class II beta chain paralogs. This class II molecule is a heterodimer consisting of an alpha (DQA) and a beta chain (DQB), both anchored in the membrane. It plays a central role in the immune system by presenting peptides derived from extracellular proteins. Class II molecules are expressed in antigen presenting cells (APC: B lymphocytes, dendritic cells, macrophages). The beta chain is approximately 26-28 kDa and it contains six exons. Exon 1 encodes the leader peptide, exons 2 and 3 encode the two extracellular domains, exon 4 encodes the transmembrane domain and exon 5 encodes the cytoplasmic tail. | Patients with MG and controls has revealed negative associations of DQB1*06 (*P*＜0.05) with the disease.And DQB1*02(*P*＜0.05) indicated increased frequencies among patients with thymic hyperplasia versus patients without hyperplasia or controls (p-values were corrected multiplying by the number of comparisons). | Negative associations or Positive association | sequence-specific oligonucleotide probes | 49 MG patients /160 HC | 14700596 | 2004[^9^](#_ENREF_9) |
|  |  |  | DQB1*0301(*P=*0.000) had a highly significant association in all MG patients compared to the controls. DQB1*0601(*P=*0.001) were less frequent in patients compared to the controls, confirming the protective effect of these alleles for MG. Patients with thymomatous MG were positively associated with DQB1 *0604 (*P=* 0.001) as compared to MG patients without thymoma. | Positive association or negative associations | PCR-SSP | 84 MG patients /293 HC | 21917268 | 2012[^12^](#_ENREF_12) |
| HLA-DRB1 | 3123 | HLA-DRB1 belongs to the HLA class II beta chain paralogs. The class II molecule is a heterodimer consisting of an alpha (DRA) and a beta chain (DRB), both anchored in the membrane. It plays a central role in the immune system by presenting peptides derived from extracellular proteins. Class II molecules are expressed in antigen presenting cells (APC: B lymphocytes, dendritic cells, macrophages). The beta chain is approximately 26-28 kDa. It is encoded by 6 exons. Exon one encodes the leader peptide; exons 2 and 3 encode the two extracellular domains; exon 4 encodes the transmembrane domain; and exon 5 encodes the cytoplasmic tail. | HLA-DRB1*16(*P=*0.05)and 13(*P=*0.05) were more frequent in MG group compared with controls. Both HLA-DRB1*07(*P=* 0.03) and *10 (*P=* 0.02)were reduced in MG group compared with controls. | Negative associations or Positive association | PCR-SSP | 109 MG patients/383 HC | 19490212 | 2009[^11^](#_ENREF_11) |
| IL32 | 9235 | This gene encodes a member of the cytokine family. Expression of this protein is increased after the activation of T-cells by mitogens or the activation of NK cells by IL-2. This protein induces the production of TNFalpha from macrophage cells. | Serum IL-32a levels were significantly higher in the MG patients(*P*=0.03): 460.07 ± 192.30 pg/mL in MG patients and 248.45 ±188.42 pg/mL in the healthy control group. | up-regulation | ELISA | 48 MG patients /35 HC | 21487807 | 2011[^13^](#_ENREF_13) |
| TNFRSF4 | 7293 | The protein encoded by this gene is a member of the TNF-receptor superfamily. This receptor has been shown to activate NF-kappaB through its interaction with adaptor proteins TRAF2 and TRAF5. Knockout studies in mice suggested that this receptor promotes the expression of apoptosis inhibitors BCL2 and BCL2lL1/BCL2-XL, and thus suppresses apoptosis. The knockout studies also suggested the roles of this receptor in CD4+ T cell response, as well as in T cell-dependent B cell proliferation and differentiation | Results from 36 MG patients and 28 HC revealed that more freshly isolated CD4+T-cells from MG patients expressed OX40 than cells from healthy individuals( *P* = 0.012). | up-regulation | Flow cytometry | 36 MG patients /28 HC | 16367941 | 2006[^14^](#_ENREF_14) |
| CD55 | 1604 | This gene encodes a protein involved in the regulation of the complement cascade. The encoded glycoprotein is also known as the decay-accelerating factor (DAF); binding of DAF to complement proteins accelerates their decay, disrupting the cascade and preventing damage to host cells. | -198C/G SNP was significantly more frequent among those developing extraocular muscle paresis associated with MG(*P*= 0.0003,odds ratio= 8.6)than normal controls | up-regulation | sequencing | 139 MG patients /167 HC | 19675582 | 2010[^15^](#_ENREF_15) |
| IL2 | 3558 | The protein encoded by this gene is a secreted cytokine that is important for the proliferation of T and B lymphocytes. The targeted disruption of a similar gene in mice leads to ulcerative colitis-like disease, which suggests an essential role of this gene in the immune response to antigenic stimuli. | peripheral blood mononuclear IL-2 production in MG was significiently higher than control patients(1110±992 vs 53±295 pg/ml,respectively; *P*＜0.001) | up-regulation | ELISA | 75 MG patients /48 HC | 12646760 | 2003[^16^](#_ENREF_16) |
| IL17A | 3605 | The protein encoded by this gene is a proinflammatory cytokine produced by activated T cells. This cytokine regulates the activities of NF-kappaB and mitogen-activated protein kinases. High levels of this cytokine are associated with several chronic inflammatory diseases including rheumatoid arthritis, psoriasis and multiple sclerosis | The concentration of IL-17 was elevated in generalized MG(10.7±10.9pg/ml) compared with ocular MG(3.4±0.8 pg/ml, P =0.017) and healthy controls(3.2 ±0.0 pg/ml , P =0.013). MG patients with AChR antibody concentrations above the median (5.72 nmol /L) had higher mean IL-17(9.97±10.60pg/ml) concentrations than those with AChR antibody concentrations below the median (3.42±0.88 pg/ml; *P*=0.032). | up-regulation | multiplexed fluorescent  bead–based immunoassay | 25 MG patients /14 HC | 21755509 | 2011[^17^](#_ENREF_17) |
| TLR4 | 7099 | The protein encoded by this gene is a member of the Toll-like receptor (TLR) family which plays a fundamental role in pathogen recognition and activation of innate immunity. They recognize pathogen-associated molecular patterns that are expressed on infectious agents, and mediate the production of cytokines necessary for the development of effective immunity. | Mean TLR4 (±SD) mRNA levels were significantly higher in thymitis (9.79±8.43) and involuted thymus (8.85 ±7.13)than in hyperplastic thymus (1.58 ±1.10) (*P*=0.0047 and 0.0026, respectively) (adjusted by Bonferroni/Dunn correction). | up-regulation | Real-Time Quantitative PCR | 37 MG patients /4 HC | 15972959 | 2005[^18^](#_ENREF_18) |
| CXCL13 | 10563 | B lymphocyte chemoattractant, is a CXC chemokine strongly expressed in the follicles of the spleen, lymph nodes, and Peyer's patches. It preferentially promotes the migration of B lymphocytes (compared to T cells and macrophages), apparently by stimulating calcium influx into, and chemotaxis of, cells expressing Burkitt's lymphoma receptor 1 (BLR-1). | The CXCL13 level was found to be higher (2.75 fold) in MG patients (N=25) than in the control subjects (N=10); In MG patients, serum CXCL13 level was found to be associated with gender (p=0.004) and age (p=0.013). | Up-regulation | ELISA;  Immunohistochemical staining;  Real-time PCR | 194 MG patients/233 HC | 20223524 | 2010[^19^](#_ENREF_19) |
| TNF | 7124 | The gene encodes a multifunctional proinflammatory cytokine that belongs to the tumor necrosis factor(TNF) superfamily. This cytokine is involved in the regulation of a wide spectrum of biological processes including cell proliferation, differentiation, apoptosis, lipid metabolism, and coagulation. Knockout studies in mice also suggested the neuroprotective function of this cytokine. | Patients with thymomatous MG had a higher frequency of TNFB*2(6/7 vs HC 32/90, *P*=0.01); Patients with EO-MG had an increased frequency of TNFB*1 (40%) compared with controls (7.8%) (*P*=0.01). | up-regulation | PCR | 47 MG patients /92 HC | 18071035 | 2007[^20^](#_ENREF_20) |
| PTPN22 | 26191 | This gene encodes of member of the non-receptor class 4 subfamily of the protein-tyrosine phosphatase family. The encoded protein is a lymphoid-specific intracellular phosphatase that associates with the molecular adapter protein CBL and may be involved in regulating CBL function in the T-cell receptor signaling pathway. | PTPN22*R620W variant(1858C/T, rs2476601) in MG: The 620W risk allele was increased in 293 nonthymoma patients without anti-titin antibodies (13.7%vs 7.4%, OR=1.97;95%CI, 1.32–2.97, *P*=0.00059) | Positive association | PCR-RFLP | 293 nonthymoma MG patients without anti-titin antibodies (total 470）/296 HC | 16437561 | 2006[^21^](#_ENREF_21) |
|  |  |  | The frequency of the T allele (W620) was significantly higher in MG patients(n=409) than in controls (n=1557) (*P* =2.7 × 10^−4^). | Positive association | MALDI-TOF  (matrix-assisted laser desorption/ionization time-of-flight mass spectrometry) | 409 MG patients /1557 HC | 18533277 | 2008[^22^](#_ENREF_22) |
|  |  |  | +1858T genotypes were significantly more prevalent in patients with MG(+) thymoma(29.1%; *P*=0.0028; OR=2.66; 95%CI 1.38-5.12) or EOMG（30.2%；*P*=0.00034； OR=2.81； 95%CI 1.58-5.00） than in the HC(13.4%). | Positive association | PCR-RFLP | 79 thymoma MG patients/129 EOMG/172 HC | 19693092 | 2009[^23^](#_ENREF_23) |
|  |  |  | There was an association of the PTPN22 1858T allele with nonthymoma MG patients with anti-titin antibodies present (T allele frequency 21% vs 11% in controls; *P=* 0.005, OR 2.1, 95% CI: 1.23–3.58) . | Positive association | Taqman SNP genotyping assay | 50 nonthymoma with anti-titin positive MG patients/379 controls | 19406179 | 2009[^24^](#_ENREF_24) |
| TGFB1 | 7040 | This gene encodes a member of the transforming growth factor beta (TGFB) family of cytokines, which are multifunctional peptides that regulate proliferation, differentiation, adhesion, migration, and other functions in many cell types. | We found significant associations between ophthalmoplegic MG with several putative functional SNPs (including two novel SNPs) that potentially alter transcription factor binding:  Compared with controls, the OP-MG phenotype showed significant associations with five known SNPs(*P*<0.05). | Positive association | PCR | 21 OP-MG/31 contols | 22458981 | 2012[^25^](#_ENREF_25) |
| IL1B | 3553 | The protein encoded by this gene is a member of the interleukin 1 cytokine family. This cytokine is produced by activated macrophages as a proprotein, which is proteolytically processed to its active form by caspase 1 (CASP1/ICE). This cytokine is an important mediator of the inflammatory response, and is involved in a variety of cellular activities, including cell proliferation, differentiation, and apoptosis. This gene and eight other interleukin 1 family genes form a cytokine gene cluster on chromosome 2. | Positive association was found with allele 2 of IL-1β TaqI RFLP in exon 5. The frequency of the genotype A2/A2 was significantly increased *P*=0.010, *P*_c_=0.030. There was a significant higher percentage of allele 2 carriage in MG than in HC (*P*=0.020, *P*c=0.040). The frequency of allele 1 was significantly lower while the frequency of allele 2 was significantly higher in patients than in HC (*P*=0.003, *P*c=0.006) (P values were corrected for the number of comparisons). | Positive association | TaqI RFLP | 107 MG patients/82 HC | 9521608 | 1998[^26^](#_ENREF_26) |
| ENOX1 | 55068 | Plasma membrane electron transport pathways are involved in functions as diverse as cellular defense, intracellular redox homeostasis, and control of cell growth and survival. Members of the ecto-NOX family, such as CNOX, or ENOX1, are involved in plasma membrane transport pathways. These enzymes exhibit both a hydroquinone (NADH) oxidase activity and a protein disulfide-thiol interchange activity in series, with each activity cycling every 22 to 26 minutes. | A region of shared homozygosity at chromosome 13q13.3-13q14.11 was found in 4 affected siblings and 1 unaffected sibling of one familial autoimmune myasthenia. A homozygous single nucleotide variant was found in the 3`-UTR of the ecto-NADH oxidase 1 gene (ENOX1).  Expression of ENOX1 decreased to about 20% of normal levels in lymphoblastoid cells from individuals homozygous for the variant and to about 50% in 2 unaffected heterozygotes. Allele-specific RT-PCR showed a 55%-60% reduction in the level of the variant transcript in heterozygous cells due to reduced mRNA stability. | New found variant | genome-wide homozygosity mapping, qRT-PCR | 7 siblings of one family with autoimmune myasthenia gravis/764 controls. | 22744667 | 2012[^27^](#_ENREF_27) |
| FCGR3B | 2215 | Fc fragment of IgG, low affinity IIIb, receptor (CD16b) | MG patients with the NA1/NA1 Fcg RIIIB genotype had the most severe MG than patients with the NA1/NA2 and the NA2/NA2 genotypes (*P=*0.01). | Positive association | PCR | 30 total MG patients/4 MG patients with NA1/NA1 genotype/49 HC | 9521619 | 1998[^28^](#_ENREF_28) |
| TAP2 | 6891 | The membrane-associated protein encoded by this gene is a member of the superfamily of ATP-binding cassette (ABC) transporters. ABC proteins transport various molecules across extra- and intra-cellular membranes. This gene is located 7 kb telomeric to gene family member ABCB2. The protein encoded by this gene is involved in antigen presentation. This protein forms a heterodimer with ABCB2 in order to transport peptides from the cytoplasm to the endoplasmic reticulum. Alternative splicing of this gene produces two products which differ in peptide selectivity and level of restoration of surface expression of MHC class I molecules. | TAP2*0101 was positively associated with MG in patients with an early onset of disease compared to patients with a late onset of disease (OR=5.11, *P*c<0.05) (adjusted by Yates’correction) . | Positive association | ARMS-PCR | 79 MG patients /155 HC | 9062975 | 1997[^29^](#_ENREF_29) |
| IL-4 | 3565 | Interleukin 4----The protein encoded by this gene is a pleiotropic cytokine produced by activated T cells. | The numbers of IL-4 mRNA-expressing cells detected in peripheral blood were higher in MG compared to patients with other neurological diseases and healthy subjects (*P*<0.0001) | Up-regulation | in situ hybridization | 33 MG patients/43 patients with other neurological diseases/25 HC | 8182116 | 1994[^30^](#_ENREF_30) |
|  |  |  | The mRNA levels of IL-4 in non-thymectomized MG patients was significantly lower than in controls(*P=*0.02) and in thymectomized MG patients(*P=* 0.03) | Down-regulation | competitive RT-PCR | 14 non-thymectomized MG patients/20 thymectomized MG patients/42 HC | 10809941 | 2000[^31^](#_ENREF_31) |
| CNTFR | 1271 | This gene encodes a member of the type 1 cytokine receptor family. The encoded protein is the ligand-specific component of a tripartite receptor for ciliary neurotrophic factor, which plays a critical role in neuronal cell survival, differentiation and gene expression. Binding of ciliary neurotrophic factor to the encoded protein recruits the transmembrane components of the receptor, gp130 and leukemia inhibitory factor receptor, facilitating signal transduction. | CNTFR gene expression was higher in muscle biopsy tissue from severely affected MG patients regardless (*P*<0.01, compared to controls). | up-regulation | RT-PCR | 44 MG patients/10 HC | 11694333 | 2001[^32^](#_ENREF_32) |
| IP-10/CXCL10 | 3627 | This gene encodes a chemokine of the CXC subfamily and ligand for the receptor CXCR3. Binding of this protein to CXCR3 results in pleiotropic effects, including stimulation of monocytes, natural killer and T-cell migration, and modulation of adhesion molecule expression. | A significant increase in IP-10 (*P*<0.005) mRNA levels in both thymus and muscle was observed in myasthenic patients compared with age-matched controls. | up-regulation | Quantitive-RT-PCR | 5 thymuses of MG patients vs 6 controls.  human muscles biopsies from 17 MG patients and four controls | 15843529 | 2005[^33^](#_ENREF_33) |
| CXCR3 | 2833 | This gene encodes a G protein-coupled receptor with selectivity for three chemokines, termed CXCL9/Mig (monokine induced by interferon-g), CXCL10/IP10 (interferon-g-inducible 10 kDa protein) and CXCL11/I-TAC (interferon-inducible T cell a-chemoattractant). Binding of chemokines to this protein induces cellular responses that are involved in leukocyte traffic, most notably integrin activation, cytoskeletal changes and chemotactic migration. | A significant increase in CXCR3 (*P*<0.05) mRNA levels in both thymus and muscle was observed in myasthenic patients compared with age-matched controls.CXCR3 expression in PBMC of MG patients was markedly increased in CD4+, but not in CD8+ T cells or in CD19+ B cells | up-regulation | Quantitive-RT-PCR | 5 thymuses of MG patients vs 6 controls.  human muscles biopsies from 17 MG patients and four controls PBMC from MG patients (n =6) and age-matched controls (n=7) | 15843529 | 2005[^33^](#_ENREF_33) |
| ESR1 | 2099 | This gene encodes an estrogen receptor, a ligand-activated transcription factor composed of several domains important for hormone binding, DNA binding, and activation of transcription. ERs have been shown to be involved in thymic development. | In MG patients, we found an increased expression of ER alpha on thymocytes (*P*<0.02) and both ERs on T cells (*P*<0.003) from peripheral blood mononuclear cells, indicating that the signals provided by thymic and peripheral microenvironments are distinct. | up-regulation | Realtime-PCR | Thymus study in 24 MG  Patients and 8 age-matched HC;  PBMC study in 9 MG  patients and  eight age-matched HC | 15661863 | 2005[^34^](#_ENREF_34) |
| PRSS16 | 10279 | This gene encodes a serine protease expressed exclusively in the thymus. It is thought to play a role in the alternative antigen presenting pathway used by cortical thymic epithelial cells during the positive selection of T cells. | Thymi from myasthenia gravis (MG) patients with thymoma showed a marked decrease in the expression of the isoforms of PRSS16 (*P* = 0.007 for PRSS16-1, *P*=0.0404 for PRSS16-4 and *P* =0.006 for PRSS16-5). | down-regulation | RT-PCR | 5 MG TFH patients/7 MG thymoma patients/6 samples from HC | 15592422 | 2005[^35^](#_ENREF_35) |
| TRB@ ( sjTREC) | 6957 | T cell receptors recognize foreign antigens which have been processed as small peptides and bound to MHC molecules at the surface of antigen presenting cells (APC). Each T cell receptor is a dimer consisting of one alpha and one beta chain or one delta and one gamma chain. | SjTREC levels were significantly raised in the thymus of patients with thymoma compared to MG patients with involuted thymus (*P*=0.01). | up-regulation | PCR | 34 MG patients/4 normal childrens | 14592884 | 2003[^36^](#_ENREF_36) |
| MMP2 | 4313 | MMPs are a group of zinc-dependent proteases with a wide range of substrates, including extracellular matrix components, cytokines, receptors and cell motility factors. They are recognized as the main proteolytic enzyme group involved in remodeling the extracellular matrix and modifying cell-cell and cell-matrix interactions .  Proteins of the MMP family are involved in the breakdown of extracellular matrix in normal physiological processes, such as embryonic development, reproduction, and tissue remodeling, as well as in disease processes, such as arthritis and metastasis. This gene encodes an enzyme which degrades type IV collagen, the major structural component of basement membranes. The enzyme plays a role in endometrial menstrual breakdown, regulation of vascularization and the inflammatory response. The gene is part of a cluster of MMP genes which localize to chromosome 11q22.3 | The MG patients had increased serum levels of MMP2 (median values 200.7 vs. 159.7 ng/ml, *P*<0.001) compared to controls. | up-regulation | ELISA | 129 MG patient/50 HC | 21212676 | 2011[^37^](#_ENREF_37) |
| MMP9 | 4318 |  | The MG patients had increased serum levels of MMP9 (median values 629.6 vs. 386.4 ng/ml, *P*<0.001) compared to controls. | up-regulation | ELISA | 129 MG patient/50 HC | 21212676 | 2011[^37^](#_ENREF_37) |
| MMP3 | 4314 |  | The subgroup of male patients had increased MMP3 concentration (*P=*0.001). | up-regulation | ELISA | 129 MG patient/90 HC | 21212676 | 2011 |
|  |  |  | The number of MMP-3 positive patients in the SPMG group was significantly higher than the number of MMP-3 positive controls (*P*<0.01). Mean MMP-3 concentration for the 16 MMP-3 positive SPMG patients was significantly higher than mean MMP-3 in the four MMP-3 positive controls (*P*<0.003). Also the mean MMP-3 concentration of the 18 MMP-3 positive SPMG and SNMG patients combined was significantly higher than mean MMP-3 in the four MMP-3 positive controls (*P*<0.001). | up-regulation | ELISA | 116 MG patients/96 SPMG patients/20 SNM G patients/90 HC | 18262287 | 2008[^38^](#_ENREF_38) |
| KCNA4 | 3739 | Potassium channels represent the most complex class of voltage-gated ion channels from both functional and structural standpoints. Their diverse functions include regulating neurotransmitter release, heart rate, insulin secretion, neuronal excitability, epithelial electrolyte transport, smooth muscle contraction, and cell volume. It belongs to the A-type potassium current class, the members of which may be important in the regulation of the fast repolarizing phase of action potentials in heart and thus may influence the duration of cardiac action potential. | Eleven of 61 MG sera immunoprecipitated a rhabdomyosarcoma-specific 70-kDa protein, which was identified as the voltage-gated K+ channel 1.4 (Kv1.4). when compared the clinical features of MG with anti-Kv1.4 antiboday with MG without this antibody, it showed that anti-Kv1.4 antibody was associated with severe form of MG, including bulbar involvement (*P*<0.0001), myasthenic crisis(*P=*0.006), thymoma (*P*<0.0001), myocarditis, and QT prolongation on ECG (*P=*0.0009). Kv1.4 mRNA was detected in all three thymoma tissues and one of two thymic hyperplasia tissues from MG patients. | up-regulation | Immunoprecipitation assay to find the autoantigens; affinity-purification and immunoblots to identify the autoantigens(Kv 1.4 protein);  PCR shows the expression of Kv1.4 mRNA in thymus. | 11 MG patients with the anti -Kv1.4 antibody and 50 MG  patients without the anti body | 16182377 | 2005[^39^](#_ENREF_39) |
| TNFSF13B | 10673 | tumor necrosis factor (ligand) superfamily, member 13b---The protein encoded by this gene is a cytokine that belongs to the tumor necrosis factor (TNF) ligand family. This cytokine is expressed in B cell lineage cells, and acts as a potent B cell activator. It has been also shown to play an important role in the proliferation and differentiation of B cells. | BAFF levels in patients with autoimmune MG were significantly higher than those in healthy subjects (P <0.001) and higher than those in patients with MS (P<0.001) and ALS (P =0.050). | up-regulation | Enzyme-linked immunosorbent assay | 43 MG patients/ 48 healthy subjects/ 3 patients with ALS/25 patients with MS | 18852352 | 2008[^40^](#_ENREF_40) |
|  |  |  | Serum BAFF levels were 562.03 ± 269.30 pg/mL in the healthy group and 747.59 ± 544.96 pg/mL in MG patients, which were significantly higher in the MG patients (*P=*0.04). | up-regulation | quantitative sandwich enzyme immunoassay | 40 MG patients /30 HC | 18586330 | 2008[^40^](#_ENREF_40) |
| AGER | 177 | The advanced glycosylation end product (AGE) receptor encoded by this gene is a member of the immunoglobulin superfamily of cell surface receptors. It is a multiligand receptor, and besides AGE, interacts with other molecules implicated in homeostasis, development, and inflammation, and certain diseases. | The levels of sRAGE significantly reduced in patients with MG compared to healthy volunteers (sRAGE [pg/ml] 927.2 ± 80.8 vs.1400.1 ± 92.4; *P*< 0.001). The actively secreted esRAGE was significantly lower in serum of patients with MG compared to controls (esRAGE [pg/ml] 273.5 ±24.6 vs. 449.0 ± 22.4; *P*< 0.001) (adjusted by Tukey correction). | down-regulation | ELISA | 42 MG patients /36 HC | 22405771 | 2012[^41^](#_ENREF_41) |
| NGF | 4803 | This gene is a member of the NGF-beta family and encodes a secreted protein which homodimerizes and is incorporated into a larger complex. This protein has nerve growth stimulating activity and the complex is involved in the regulation of growth and the differentiation of sympathetic and certain sensory neurons. | The level of NGF in the thymus of normal human subjects ranges from 155 to 200 pg/gr tissue (mean 185 pg/gr) which is significantly lower as compared to the mean concentration of NGF found in the thymus of patients affected by MG (432 pg/gr).(*P*<0.01) | up-regulation | ELISA | 13 thymuses from MG patients/5 normal thymuses | 15763921 | 2004[^42^](#_ENREF_42) |
| IGF1 | 3479 | The protein encoded by this gene is similar to insulin in function and structure and is a member of a family of proteins involved in mediating growth and development. It is known to increase the numbers of lymphocytes in the thymus and spleen, and enhance their function via greater lymphocyte generation and/or survival. IGF-I targets some progenitor cells of the immune sys-tem, including promyeloid, pro-B, and pro-T cells, promotes passage through the cell cycle, impairs cellular death, and increases differentiation to a more mature phenotype. | Quantitative analysis carried out in comparable thymic sections of each thymus from both patients and controls revealed an increase in the distribution of IGF-I positive EC in the pathological thymi (MG) compared to the controls: cortex 23.33±3.33 vs 10.00±1.29 (*P*<0.005); medulla 40.83±7.23 vs 20.00±4.47 (*P*<0.05). | up-regulation | Immunohistochemistry and quantitative evaluation | 14 thymus specimens of MG patients/6 specimens from normal thymus glands. | 18254780 | 2008[^43^](#_ENREF_43) |
| IGF1R | 3480 | This receptor binds insulin-like growth factor with a high affinity. It has tyrosine kinase activity. The insulin-like growth factor I receptor plays a critical role in transformation events. It is highly overexpressed in most malignant tissues where it functions as an anti-apoptotic agent by enhancing cell survival. | An increase in the distribution of IGF-IR positive EC in pathological thyme (MG) compared to controls was evident: cortex 19.16±3.00 vs 7.50 ±1.11 (*P*<0.005); medulla 41.66 ±2.47 vs 15.83±1.53 (*P*<0.001) | up-regulation | Immunohistochemistry and quantitative evaluation | 14 thumus specimens of MG patients/6 normal specimens. | 18254780 | 2008[^43^](#_ENREF_43) |
| TFRC | 7037 | CD71 is a transferring receptor which is composed of two identical glycoproteins that form a disulfide linked dimer. | There was a difference in the percentage of B-cells which expressed CD71 in the peripheral blood. The mean percentage ±SD of the CD20/CD71 B cell population was 23.1±13.5 for MG patiens and 15.6±14.7 for controls; the medians were 20.7 and 10.8, respectively. The difference was statistically significant (*P*<0.05, Mann-Whitney test). | up-regulation | Immunogluorescent and flow cytometry | 47 MG patients/35 HC | 11706095 | 2001[^44^](#_ENREF_44) |
| Bcl-2 | 596 | This gene encodes an integral outer mitochondrial membrane protein that blocks the apoptotic death of some cells such as lymphocytes. Constitutive expression of BCL2, such as in the case of translocation of BCL2 to Ig heavy chain locus, is thought to be the cause of follicular lymphoma. Two transcript variants, produced by alternate splicing, differ in their C-terminal ends. | The percentage of Bcl-2—positive cells in the medulla was significantly greater in the hyperplastic MG thymuses than in the control thymuses (63.5±6.0% vs 30.2±2.3%, *P*<0.05). | up-regulation | Immunocytochemistry, quantitive analysis of Bcl-2—positive cells | Tymic tissure are from 10 MG patients and 4 HC. | 8619530 | 1996[^45^](#_ENREF_45) |
|  |  |  | The frequency of Bcl-2^+^ GC B cells in the MG thymus was significantly higher than that in control tonsils (54.3±16.2 versus 20.6±8.0 %, *P*<0.0001, unpaired t-test). | up-regulation | Flow cytometry | 14 thymuses from generalized MG/8 control tonsils from HC | 9130628 | 1997[^46^](#_ENREF_46) |
|  |  |  | Bcl-2 expression in medulla was higher in patients with MG compared to controls (*P*<0.001). Bcl-2 expression is correlated reversibly with MG stage (*P*<0.001) (Bonferroni correction). | up-regulation | Immunohistochemistry and In situ hybridization for mRNA detection | 38 thymuses from MG patients/5 thymuses from controls | 11574213 | 2001[^47^](#_ENREF_47) |
| BAX | 581 | The protein encoded by this gene belongs to the BCL2 protein family. BCL2 family members form hetero- or homodimers and act as anti- or pro-apoptotic regulators that are involved in a wide variety of cellular activities. This protein forms a heterodimer with BCL2, and functions as an apoptotic activator. This protein is reported to interact with, and increase the opening of, the mitochondrial voltage-dependent anion channel (VDAC), which leads to the loss in membrane potential and the release of cytochrome c. The expression of this gene is regulated by the tumor suppressor P53 and has been shown to be involved in P53-mediated apoptosis. | Bax expression was higher in patients with MG compared to controls (*P*<0.001). In addition, there was a trend for increased bax expression toward advanced MG stage (*P*<0. 001) (Bonferroni correction). | up-regulation | Immunohistochemistry and In situ hybridization for mRNA detection | 38 thymuses from MG patients/5 thymuses from controls | 11574213 | 2001[^47^](#_ENREF_47) |
| MKI67 | 4288 | This gene encodes a nuclear protein that is associated with and may be necessary for cellular proliferation. This antigen recognizes an epitope on a nuclear antigen in cell cycling and labels cells that are in phase S of the cell cycle. The proportion of Ki67-labeled cells in a given cell population provides a measure of the growth fraction. | Ki67 expression was higher in patients with MG compared to controls (*P*<0.001). There was a trend for increased Ki67 expression both in the medulla and in the cortex toward advanced MG stage (*P*<0.001 for medulla or cortex) (Bonferroni correction). | up-regulation | Immunohistochemistry and In situ hybridization for mRNA detection | 38 thymuses from MG patients/5 thymuses from controls | 11574213 | 2001[^47^](#_ENREF_47) |
| IL18 | 3606 | interleukin 18 (interferon-gamma-inducing factor)--- The protein encoded by this gene is a proinflammatory cytokine that augments natural killer cell activity in spleen cells, and stimulates interferon gamma production in T-helper type I cells. | IL-18 serum levels in patients with MG were elevated above the control level (362±45 pg/mL vs 64±13 pg/mL; *P*<0.0001). IL-18 levels exhibited a clear further increase in generalized MG when compared to ocular myasthenia (436±61 pg/mL vs 224±49 pg/mL; *P=*0.0096). | up-regulation | ELISA | 78 MG patients/33 neurologic patients without evidence for any inflammatory, autoimmune, infectious, or malignant disease condition | 12136075 | 2002[^48^](#_ENREF_48) |
| CTLA-4 | 1493 | This gene is a member of the immunoglobulin superfamily and encodes a protein which transmits an inhibitory signal to T cells. Mutations in this gene have been associated with insulin-dependent diabetes mellitus, Graves disease, Hashimoto thyroiditis, celiac disease, systemic lupus erythematosus, thyroid-associated orbitopathy, and other autoimmune diseases. | There was a clear trend toward greater MG frequencies in the three 318+49 genotypes (TA/TA, CA/TA, and CA/CA) with +49A/A. The allelic frequency of the CTLA4 high+49A was also significantly greater(67.7%) in MG than in MG thymoma patients (52.2%; *P*= 0.015). | up-regulation | PCR-RFLP | 208 MG patients/173 HC | 16178018 | 2005[^49^](#_ENREF_49) |
|  |  |  | The frequency of the TC genotype at position ) 1772 was higher in MG patients than in healthy individuals *P*=0.049) | up-regulation | RFLP  Cells and cell cultures  RT-PCR | 165 MG patients/148 HC | 18088253 | 2008[^50^](#_ENREF_50) |
| CHRNA1 | 1134 | The muscle acetylcholine receptor consiststs of 5 subunits of 4 different types: 2 alpha isoforms and 1 each of beta, gamma, and delta subunits. This gene encodes an alpha subunit that plays a role in acetlycholine binding/channel gating. | Genotyping of rs16862847 in the French data set showed an increased minor allele frequency in myasthenia gravis patients with an age at disease onset in the lower quartile (odds ratio, 2.35; 95% CI: 1.31–4.23; *P*=0.0049) | up-regulation | quantitative RT–PCR | 180 MG patients /151 HC | 17687331 | 2007[^51^](#_ENREF_51) |
| CHRNB1 | 1140 | The muscle acetylcholine receptor is composed of five subunits: two alpha subunits and one beta, one gamma, and one delta subunit. This gene encodes the beta subunit of the acetylcholine receptor. Mutations in this gene are associated with slow-channel congenital myasthenic syndrome. | Regarding nAChRβ-subunit mRNA expression, note the significant increase in both severely affected patients (*P*<0.002) and moderately affected patients (*P*<0.02) relative to controls.  There was a significant increase in expression of nAChR δ- and nAChR ε-subunit mRNA in severely affected patients (*P*<0.01 and  *P*<0.006, respectively). | up-regulation | quantitative PCR;  Northern blotting | 22 MG patients /7 HC(β-subunit)  18 MG patients /7 HC(δ-subunit)  17 MG patients /5 HC(ε-subunit) | 9649579 | 1998[^52^](#_ENREF_52) |
| CHRND | 1144 | The acetylcholine receptor of muscle has 5 subunits of 4 different types: 2 alpha and 1 each of beta, gamma and delta subunits. | Nonthymoma patients showed a modest but significant increase in the frequency of CHRND_MS*268, compared with healthy control subjects (14.9 vs 8.9%, OR=1.78, *P=* 0.038).  Patients negative for anti-titin antibodies showed a significant association with the allele 268, with an odds ratio of 2.07 (*P*=0.017). | up-regulation | PCR | 444 MG patients /168 HC | 14735155 | 2004[^53^](#_ENREF_53) |
| CHRNE | 1145 | Acetylcholine receptors at mature mammalian neuromuscular junctions are pentameric protein complexes composed of four subunits in the ratio of two alpha subunits to one beta, one epsilon, and one delta subunit. The acetylcholine receptor changes subunit composition shortly after birth when the epsilon subunit replaces the gamma subunit seen in embryonic receptors. | We found that the levels ofβδε-subunit mRNA coding for the adult nAChR were increased in sternocleidomastoid muscle samples of severely affected MG patients, matching our previous data on the α-subunit. Messenger levels were highly variable in MG patients but not in controls, pointing to individual factors involved in the regulation of nAChR genes. | up-regulation | quantitative PCR  Northern blotting | 27 MG patients /10 HC | 9649579 | 1998[^52^](#_ENREF_52) |
| CTSL2 | 1515 | The protein encoded by this gene, a member of the peptidase C1 family, is a lysosomal cysteine proteinase that may play an important role in corneal physiology. This gene is expressed in colorectal and breast carcinomas but not in normal colon, mammary gland, or peritumoral tissues, suggesting a possible role for this gene in tumor processes. | An association was seen between the early-onset MG subgroup and rs4361859 situated~23 kb upstream of the gene (45% in cases vs 31% in controls; OR 1.82, 95% CI 1.07–3.12,*P*=0.03;) a two-SNP haplotype analysis (rs4361859 and rs4743056) showed an association equal to that observed for rs4361859 alone (45% in cases vs 31.1% in controls; OR=1.81; 95% CI 1.06 –3.1, *P=*0.03) (permutation testing), | down-regulation | RT-PCR | 83 MG patients /244 HC | 17869649 | 2007[^54^](#_ENREF_54) |
| FCGR2A | 2212 | This gene encodes one member of a family of immunoglobulin Fc receptor genes found on the surface of many immune response cells. The protein encoded by this gene is a cell surface receptor found on phagocytic cells such as macrophages and neutrophils, and is involved in the process of phagocytosis and clearing of immune complexes. | Fc g RIIa genotypes were differentially distributed among 107 MG patients as compared to 239 HC, with a relative increase of the FcgRIIa-R/R131 genotype (Odds ratio 2.4, 95% confidence interval 1.4 – 3.9). | up-regulation | PCR  modified alleles pecific reaction | 107 MG patients /239 HC | 14597109 | 2003[^55^](#_ENREF_55) |
|  |  |  | Patients with thymomatous MG had a higher frequency of TNFB*2 (*P*=0.01) and FCGR2A 131H/H(*P*=0.05) | up-regulation | PCR-RFLP | 47 MG patients /92 HC | 18071035 | 2007[^20^](#_ENREF_20) |
| APOE | 348 | Chylomicron remnants and very low density lipoprotein (VLDL) remnants are rapidly removed from the circulation by receptor-mediated endocytosis in the liver. Apolipoprotein E, a main apoprotein of the chylomicron, binds to a specific receptor on liver cells and peripheral cells. ApoE is essential for the normal catabolism of triglyceride-rich lipoprotein constituents. The APOE gene is mapped to chromosome 19 in a cluster with APOC1 and APOC2. | A significant association of Apo-E4 allele with AChR-antibody positive patients was observed (*P*=0.007).  Among seropositive patients, a significant association was seen between female gender and Apo-E4 allele (*P*=0.023). | up-regulation | PCR-RFLP | 120 MG patients /120 HC | 20644276 | 2010[^56^](#_ENREF_56) |
| ADRB2 | 154 | This gene encodes beta-2-adrenergic receptor which is a member of the G protein-coupled receptor superfamily. This receptor is directly associated with one of its ultimate effectors, the class C L-type calcium channel Ca(V)1.2. This receptor-channel complex also contains a G protein, an adenylyl cyclase, cAMP-dependent kinase, and the counterbalancing phosphatase, PP2A. The assembly of the signaling complex provides a mechanism that ensures specific and rapid signaling by this G protein-coupled receptor. This gene is intronless. | Increased prevalence of homozygosity for Arg16 in MG patients with generalized MG (*P=*0.0022, *P*c=0.0132, OR=3.6. 95%CI=1.52-8.54). | up-regulation | allele-specific PCR | 145 MG patients /96 HC | 10606977 | 2000[^57^](#_ENREF_57) |
|  |  |  | The frequency of homozygosity for Gly16 was lower in MG patients than in healthy individuals (*P=*0·0079, *P*c=0·0474, OR=0.45.95%CI=0.26-0.81) ) (Yates’ correction). | Down-regulation | allele-specific PCR | 145 MG patients /96 HC | 10606977 | 2000[^57^](#_ENREF_57) |
| IFNG | 3458 | This gene encodes a member of the type II interferon family. The protein encoded is a soluble cytokine with antiviral, immunoregulatory and anti-tumor properties and is a potent activator of macrophages. | IFNG +874T carriers were less frequent in MG, in patients with anti-acetylcholine receptor (AChR) (63%) and anti-titin (56.2%) antibodies compared with HC (*P=*0.01 for all, OR: 0.5, 0.5, and 0.4, respectively). The presence of thymoma was also associated with lower frequency of IFNG +874T allele (*P=*0.018, OR=0.34) (Bonferroni correction). | Down-regulation | PCR–sequence-specific | 115 MG patients /204 HC | 17509455 | 2007[^58^](#_ENREF_58) |
| IL–10 | 3586 | The protein encoded by this gene is a cytokine produced primarily by monocytes and to a lesser extent by lymphocytes. This cytokine has pleiotropic effects in immunoregulation and inflammation. It down-regulates the expression of Th1 cytokines, MHC class II Ags, and costimulatory molecules on macrophages. It also enhances B cell survival, proliferation, and antibody production. This cytokine can block NF-kappa B activity, and is involved in the regulation of the JAK-STAT signaling pathway. Knockout studies in mice suggested the function of this cytokine as an essential immunoregulator in the intestinal tract. | MG patients had a significantly higher frequency of the ACC/ACC haplotype (12.5%) when compared to controls (3.4%) (*P*=0.05) the MG patient subgroup with thymoma had a significantly higher frequency of ACC/ACC (21.4%) than controls (*P* =0.03), as had the LO-MG patients (20%) (*P*=0.02). A higher frequency of the ATA/ATA haplotype was observed in EO-MG patients (19.2%) compared to controls (*P*=0.02). MG patients had a lower than expected ACC/ATA frequency (6.3%) compared to controls (19.5%) (*P*=003), and LO-MG patients had a lower frequency of ATA/GCC (5.0%) compared to other MG patients (*P*=0.0 06). | up-regulation | PCR | 64 MG patients /87 HC | 19299022 | 2009[^59^](#_ENREF_59) |
|  |  |  | In the patients with MG, the IL-10 genotype ACC/ACC occurred with significantly increased frequency EOMG (*P*=0.05). | up-regulation | RFLP | 47 MG patients /92 HC | 18071035 | 2007[^20^](#_ENREF_20) |
| IL-1A | 3552 | The protein encoded by this gene is a member of the interleukin 1 cytokine family. This cytokine is a pleiotropic cytokine involved in various immune responses, inflammatory processes, and hematopoiesis. This cytokine is produced by monocytes and macrophages as a proprotein, which is proteolytically processed and released in response to cell injury, and thus induces apoptosis. This gene and eight other interleukin 1 family genes form a cytokine gene cluster on chromosome 2. | the IL-1A-889 CC genotype was associated with early disease onset (*P*=0.0044) in the whole MG group and the subgroup of CC males developed MG about 18 years earlier than males carrying other IL-1A-889 genotypes (*P*=0.022) (Bonferroni correction). | Positive associated | RFLP | 421 MG patients /995 HC | 11777547 | 2002[^60^](#_ENREF_60) |
| IL2RB | 3560 | The interleukin 2 receptor, which is involved in T cell-mediated immune responses, is present in 3 forms with respect to ability to bind interleukin 2. The low affinity form is a monomer of the alpha subunit and is not involved in signal transduction. The intermediate affinity form consists of an alpha/beta subunit heterodimer, while the high affinity form consists of an alpha/beta/gamma subunit heterotrimer. Both the intermediate and high affinity forms of the receptor are involved in receptor-mediated endocytosis and transduction of mitogenic signals from interleukin 2. The protein encoded by this gene represents the beta subunit and is a type I membrane protein. | A significant difference was found in the distribution of the rs743777 polymorphism haplotypes (*P=*0.01). | Positive associated | TaqMan Allelic Discrimination Assays | 146 MG patients /291 HC | 20728947 | 2010[^61^](#_ENREF_61) |
| IL4R | 3566 | This gene encodes the alpha chain of the interleukin-4 receptor, a type I transmembrane protein that can bind interleukin 4 and interleukin 13 to regulate IgE production. The encoded protein also can bind interleukin 4 to promote differentiation of Th2 cells. A soluble form of the encoded protein can be produced by proteolysis of the membrane-bound protein, and this soluble form can inhibit IL4-mediated cell proliferation and IL5 upregulation by T-cells. Allelic variations in this gene have been associated with atopy, a condition that can manifest itself as allergic rhinitis, sinusitus, asthma, or eczema. Polymorphisms in this gene are also associated with resistance to human immunodeficiency virus type-1 infection. | The rare GG genotype, which results in 2 copies of the V75 allele, was significantly associated with MG (*P=*0.023, ExB: 1.78, CI: 1.08–3.08) (Bonferroni correction). | up-regulation | TaqMan Allelic Discrimination Assays | 260 MG patients /299 HC | 22119518 | 2012[^62^](#_ENREF_62) |
| LGALS1 | 3956 | The galectins are a family of beta-galactoside-binding proteins implicated in modulating cell-cell and cell-matrix interactions. This gene product may act as an autocrine negative growth factor that regulates cell proliferation. | A significant difference was found in the distribution of the rs4820293/rs743777 polymorphism haplotypes (*P=*0.01). rs4820293 is significant for MG (*P=*0.022), for thymus pathology (*P*=0.037), and for AChR positivity (*P=*0.019). | Positive association | TaqMan Allelic Discrimination Assays | 146 MG patients /291 HC | 20728947 | 2010[^61^](#_ENREF_61) |
| HSP90B1 | 7184 | This gene encodes a member of a family of adenosine triphosphate(ATP)-metabolizing molecular chaperones with roles in stabilizing and folding other proteins. The encoded protein is localized to melanosomes and the endoplasmic reticulum. Expression of this protein is associated with a variety of pathogenic states, including tumor formation. There is a microRNA gene located within the 5' exon of this gene. There are pseudogenes for this gene on chromosomes 1 and 15. | The 90-kDa autoantigens GRP94 were recognized in 24 (7.1%) of 336 MG sera, but in none of the diseases control or healthy control sera (*P*=0.01). | down-regulation | ELISA  immunoprecipitation assay | 341 MG patients/28 patients with Duchenne muscular dystrophy (DMD), 20 patients with thymoma without M G and 28 healthy volunteers. | 21774995 | 2011[^63^](#_ENREF_63) |
| CA 3 | 761 | Carbonic anhydrase III (CAIII) is a member of a multigene family (at least six separate genes are known) that encodes carbonic anhydrase isozymes. These carbonic anhydrases are a class of metalloenzymes that catalyze the reversible hydration of carbon dioxide and are differentially expressed in a number of cell types. The expression of the CA3 gene is strictly tissue specific and present at high levels in skeletal muscle and much lower levels in cardiac and smooth muscle. | Densitograms of MG patients (1.04±0.18) were significantly lower than that of normal controls (1.27±0.21, *P=*0.001) and OMD patients (1.21± 0.15, *P=* 0.05) | down-regulation | Immunoblot assay  Immunohistochemistry | 28 MG patients/24 HC | 19301202 | 2009[^64^](#_ENREF_64) |
| IL-12B | 3593 | This gene encodes a subunit of interleukin 12, a cytokine that acts on T and natural killer cells, and has a broad array of biological activities. This cytokine is expressed by activated macrophages that serve as an essential inducer of Th1 cells development. This cytokine has been found to be important for sustaining a sufficient number of memory/effector Th1 cells to mediate long-term protection to an intracellular pathogen. | MG patients had reduced levels of IL-12 (*P*<0.01) in sera compared to controls. | down-regulation | ELISA  Flow cytometry techniques | 75 MG patients /50 HC | 18054287 | 2008[^65^](#_ENREF_65) |
| IL12A | 3592 | This gene encodes a subunit of a cytokine that acts on T and natural killer cells, and has a broad array of biological activities. This cytokine is required for the T-cell-independent induction of interferon (IFN)-gamma, and is important for the differentiation of both Th1 and Th2 cells. The responses of lymphocytes to this cytokine are mediated by the activator of transcription protein STAT4. | MG patients had reduced levels of IL-12 (*P*<0.01) in sera compared to controls. | down-regulation | ELISA  Flow cytometry techniques | 75 MG patients /50 HC | 18054287 | 2008[^65^](#_ENREF_65) |
| TNIP1 | 10318 | TNFAIP3 interacting protein 1, this gene encodes an A20-binding protein which plays a role in autoimmunity and tissue homeostasis through the regulation of nuclear factor kappa-B activation. Mutations in this gene have been associated with psoriatic arthritis, rheumatoid arthritis, and systemic lupus erythematosus. Multiple transcript variants encoding different isoforms have been found for this gene. | Researchers have found the imputed coding variant (rs2233290) at position 151 (Pro-Ala) in the TNFAIP3-interacting protein 1,TNIP1 , confers even strong risk in EOMG(p = 3.2 *10^-10^). Another SNP( rs4958881) in TNIP1 also shows strong positive association with EOMG (meta-analysis, p^c^=2.5*10^-10^). | Positive association | 2-stage genome-wide association study | 649 EOMG/~2600 HC | 23055271 | 2012[^66^](#_ENREF_66) |
| STAT4 | 6775 | signal transducer and activator of transcription 4. The protein encoded by this gene is a member of the STAT family of transcription factors. In response to cytokines and growth factors, STAT family members are phosphorylated by the receptor associated kinases, and then form homo- or heterodimers that translocate to the cell nucleus where they act as transcription activators. This protein is essential for mediating responses to IL12 in lymphocytes, and regulating the differentiation of T helper cells. Mutations in this gene may be associated with systemic lupus erythematosus and rheumatoid arthritis. | Researchers have found the SNP(rs6752770) in STAT4 shows strong positive association with EOMG (meta-analysis, p^c^=5.17*10^-4^). | Positive association | 2-stage genome-wide association study | 649 EOMG/~2600 HC | 23055271 | 2012[^66^](#_ENREF_66) |
| IKZF1 | 10320 | IKAROS family zinc finger 1 (Ikaros); This gene encodes a transcription factor that belongs to the family of zinc-finger DNA binding proteins associated with chromatin remodeling. The expression of this protein functions as a regulator of lymphocyte differentiation. All isoforms share a common C-terminal domain, which contains two zinc finger motifs that are required for hetero- or homodimerization, and for interactions with other proteins. Overexpression of some dominant-negative isoforms have been associated with B-cell malignancies, such as acute lymphoblastic leukemia (ALL). | Researchers have found the SNP(rs1026421) in IKZF1 shows strong positive association with EOMG (meta-analysis, p^c^=3.46*10^-4^). | Positive association | 2-stage genome-wide association study | 649 EOMG/~2600 HC | 23055271 | 2012[^66^](#_ENREF_66) |
| IRF5 | 3663 | interferon regulatory factor 5. This gene encodes a member of the interferon regulatory factor (IRF) family, a group of transcription factors with diverse roles, including virus-mediated activation of interferon, and modulation of cell growth, differentiation, apoptosis, and immune system activity. Members of the IRF family are characterized by a conserved N-terminal DNA-binding domain containing tryptophan (W) repeats. | Researchers have found the SNP(rs729302) in IRF5 shows strong positive association with EOMG (meta-analysis, p^c^=2.45*10^-5^). | Positive association | 2-stage genome-wide association study | 649 EOMG/~2600 HC | 23055271 | 2012[^66^](#_ENREF_66) |
| NKX2-3 | 159296 | NK2 homeobox 3. This gene encodes a homeodomain-containing transcription factor. The encoded protein is a member of the NKX family of homeodomain transcription factors. Studies of similar proteins in mouse and rat have indicated a potential role in cellular differentiation. | Researchers have found the SNP(rs11190140) in NKX2-3 shows strong positive association with EOMG (meta-analysis, p^c^=4.61*10^-4^). | Positive association | 2-stage genome-wide association study | 649 EOMG/~2600 HC | 23055271 | 2012[^66^](#_ENREF_66) |
| ORMDL3 | 94103 | ORM1-like 3 (S. cerevisiae). | Researchers have found the SNP(rs9303277) in ORMDL3 shows strong positive association with EOMG (meta-analysis, p^c^=1.25*10^-5^). | Positive association | 2-stage genome-wide association study | 649 EOMG/~2600 HC | 23055271 | 2012[^66^](#_ENREF_66) |
| CD226 | 10666 | This gene encodes a glycoprotein expressed on the surface of NK cells, platelets, monocytes and a subset of T cells. It is a member of the Ig-superfamily containing 2 Ig-like domains of the V-set. The protein mediates cellular adhesion of platelets and megakaryocytic cells to vascular endothelial cells. The protein also plays a role in megakaryocytic cell maturation. | Researchers have found the SNP(rs1790588) in CD555 shows strong positive association with EOMG (meta-analysis, p^c^=3.97*10^-4^). | Positive association | 2-stage genome-wide association study | 649 EOMG/~2600 HC | 23055271 | 2012[^66^](#_ENREF_66) |
| PTTG1 | 9232 | pituitary tumor-transforming 1. The encoded protein is a homolog of yeast securin proteins, which prevent separins from promoting sister chromatid separation. It is an anaphase-promoting complex (APC) substrate that associates with a separin until activation of the APC. The gene product has transforming activity in vitro and tumorigenic activity in vivo, and the gene is highly expressed in various tumors. The gene product contains 2 PXXP motifs, which are required for its transforming and tumorigenic activities, as well as for its stimulation of basic fibroblast growth factor expression. | Researchers have found the SNP(rs2431697) in PTTG1 shows strong positive association with EOMG (meta-analysis, p^c^=1.38*10^-5^). | Positive association | 2-stage genome-wide association study | 649 EOMG/~2600 HC | 23055271 | 2012[^66^](#_ENREF_66) |
| FOXP3 | 50943 | forkhead box P3. The protein encoded by this gene is a member of the forkhead/winged-helix family of transcriptional regulators. Defects in this gene are the cause of immunodeficiency polyendocrinopathy, enteropathy, X-linked syndrome (IPEX), also known as X-linked autoimmunity-immunodeficiency syndrome. Alternatively spliced transcript variants encoding different isoforms have been identified. | Researchers found that the frequency of the FOXP3 IVS9+459 G allele was significantly lower in MG patients than in healthy controls (P=0.041). | Negative associatioin | PCR-RFLP | 118 MG/124 Healthy Controls | 23228687 | 2012[^67^](#_ENREF_67) |

Note: Red words indicated the p-value has been adjusted for multiple testing.

**Reference**

1. Vrolix K, Niks EH, Le Panse R *et al*: Reduced thymic expression of ErbB receptors without auto-antibodies against synaptic ErbB in myasthenia gravis. *J Neuroimmunol* 2011; **232:** 158-165.

2. Nagata T, Onodera H, Ohuchi M *et al*: Decreased expression of c-myc family genes in thymuses from myasthenia gravis patients. *J Neuroimmunol* 2001; **115:** 199-202.

3. Gilboa-Geffen A, Lacoste PP, Soreq L *et al*: The thymic theme of acetylcholinesterase splice variants in myasthenia gravis. *Blood* 2007; **109:** 4383-4391.

4. Berrih-Aknin S, Ruhlmann N, Bismuth J *et al*: CCL21 overexpressed on lymphatic vessels drives thymic hyperplasia in myasthenia. *Ann Neurol* 2009; **66:** 521-531.

5. Pal Z, Antal P, Srivastava SK *et al*: Non-synonymous single nucleotide polymorphisms in genes for immunoregulatory galectins: Association of galectin-8 (F19Y) occurrence with autoimmune diseases in a Caucasian population. *Biochim Biophys Acta* 2012; **1820:** 1512-1518.

6. Colombara M, Antonini V, Riviera AP *et al*: Constitutive activation of p38 and ERK1/2 MAPKs in epithelial cells of myasthenic thymus leads to IL-6 and RANTES overexpression: effects on survival and migration of peripheral T and B cells. *J Immunol* 2005; **175:** 7021-7028.

7. Casciola-Rosen L, Miagkov A, Nagaraju K *et al*: Granzyme B: evidence for a role in the origin of myasthenia gravis. *J Neuroimmunol* 2008; **201-202:** 33-40.

8. Donmez B, Ozakbas S, Oktem MA *et al*: HLA genotypes in Turkish patients with myasthenia gravis: comparison with multiple sclerosis patients on the basis of clinical subtypes and demographic features. *Hum Immunol* 2004; **65:** 752-757.

9. Fernandez-Mestre MT, Vargas V, Montagnani S, Cotua M, Ogando V, Layrisse Z: HLA class II and class I polymorphism in Venezuelan patients with myasthenia gravis. *Hum Immunol* 2004; **65:** 54-59.

10. Vandiedonck C, Raffoux C, Eymard B *et al*: Association of HLA-A in autoimmune myasthenia gravis with thymoma. *J Neuroimmunol* 2009; **210:** 120-123.

11. Hajeer AH, Sawidan FA, Bohlega S *et al*: HLA class I and class II polymorphisms in Saudi patients with myasthenia gravis. *Int J Immunogenet* 2009; **36:** 169-172.

12. Yang H, Hao J, Peng X *et al*: The association of HLA-DQA1*0401 and DQB1*0604 with thymomatous myasthenia gravis in northern Chinese patients. *J Neurol Sci* 2012; **312:** 57-61.

13. Na SJ, So SH, Lee KO, Choi YC: Elevated serum level of interleukin-32alpha in the patients with myasthenia gravis. *J Neurol* 2011; **258:** 1865-1870.

14. Xiaoyan Z, Pirskanen R, Malmstrom V, Lefvert AK: Expression of OX40 (CD134) on CD4+ T-cells from patients with myasthenia gravis. *Clin Exp Immunol* 2006; **143:** 110-116.

15. Heckmann JM, Uwimpuhwe H, Ballo R, Kaur M, Bajic VB, Prince S: A functional SNP in the regulatory region of the decay-accelerating factor gene associates with extraocular muscle pareses in myasthenia gravis. *Genes Immun* 2010; **11:** 1-10.

16. Utsugisawa K, Nagane Y, Obara D, Kondoh R, Yonezawa H, Tohgi H: Interleukin-2 production by peripheral blood mononuclear cells from patients with myasthenia gravis. *Eur Neurol* 2003; **49:** 160-163.

17. Roche JC, Capablo JL, Larrad L *et al*: Increased serum interleukin-17 levels in patients with myasthenia gravis. *Muscle Nerve* 2011; **44:** 278-280.

18. Bernasconi P, Barberis M, Baggi F *et al*: Increased toll-like receptor 4 expression in thymus of myasthenic patients with thymitis and thymic involution. *Am J Pathol* 2005; **167:** 129-139.

19. Shiao YM, Lee CC, Hsu YH *et al*: Ectopic and high CXCL13 chemokine expression in myasthenia gravis with thymic lymphoid hyperplasia. *J Neuroimmunol* 2010; **221:** 101-106.

20. Amdahl C, Alseth EH, Gilhus NE, Nakkestad HL, Skeie GO: Polygenic disease associations in thymomatous myasthenia gravis. *Arch Neurol* 2007; **64:** 1729-1733.

21. Vandiedonck C, Capdevielle C, Giraud M *et al*: Association of the PTPN22*R620W polymorphism with autoimmune myasthenia gravis. *Ann Neurol* 2006; **59:** 404-407.

22. Lefvert AK, Zhao Y, Ramanujam R, Yu S, Pirskanen R, Hammarstrom L: PTPN22 R620W promotes production of anti-AChR autoantibodies and IL-2 in myasthenia gravis. *J Neuroimmunol* 2008; **197:** 110-113.

23. Chuang WY, Strobel P, Belharazem D *et al*: The PTPN22gain-of-function+1858T(+) genotypes correlate with low IL-2 expression in thymomas and predispose to myasthenia gravis. *Genes Immun* 2009; **10:** 667-672.

24. Greve B, Hoffmann P, Illes Z *et al*: The autoimmunity-related polymorphism PTPN22 1858C/T is associated with anti-titin antibody-positive myasthenia gravis. *Hum Immunol* 2009; **70:** 540-542.

25. Akinyi MV, Dandara C, Gamieldien J, Heckmann JM: Association of transforming growth factor beta-1 (TGFB1) regulatory region polymorphisms with myasthenia gravis-related ophthalmoparesis. *J Neuroimmunol* 2012.

26. Huang D, Pirskanen R, Hjelmstrom P, Lefvert AK: Polymorphisms in IL-1beta and IL-1 receptor antagonist genes are associated with myasthenia gravis. *J Neuroimmunol* 1998; **81:** 76-81.

27. Landoure G, Knight MA, Stanescu H *et al*: A candidate gene for autoimmune myasthenia gravis. *Neurology* 2012.

28. Raknes G, Skeie GO, Gilhus NE, Aadland S, Vedeler C: FcgammaRIIA and FcgammaRIIIB polymorphisms in myasthenia gravis. *J Neuroimmunol* 1998; **81:** 173-176.

29. Hjelmstrom P, Giscombe R, Lefvert AK *et al*: TAP polymorphisms in Swedish myasthenia gravis patients. *Tissue Antigens* 1997; **49:** 176-179.

30. Link J, Navikas V, Yu M, Fredrikson S, Osterman PO, Link H: Augmented interferon-gamma, interleukin-4 and transforming growth factor-beta mRNA expression in blood mononuclear cells in myasthenia gravis. *J Neuroimmunol* 1994; **51:** 185-192.

31. Huang WX, Huang P, Fredrikson S, Pirskanen R, Hillert J: Decreased mRNA expression of TNF-alpha and IL-10 in non-stimulated peripheral blood mononuclear cells in myasthenia gravis. *Eur J Neurol* 2000; **7:** 195-202.

32. Poea S, Guyon T, Levasseur P, Berrih-Aknin S: Expression of ciliary neurotrophic factor receptor in myasthenia gravis. *J Neuroimmunol* 2001; **120:** 180-189.

33. Feferman T, Maiti PK, Berrih-Aknin S *et al*: Overexpression of IFN-induced protein 10 and its receptor CXCR3 in myasthenia gravis. *J Immunol* 2005; **174:** 5324-5331.

34. Nancy P, Berrih-Aknin S: Differential estrogen receptor expression in autoimmune myasthenia gravis. *Endocrinology* 2005; **146:** 2345-2353.

35. Luther C, Wienhold W, Oehlmann R, Heinemann MK, Melms A, Tolosa E: Alternatively spliced transcripts of the thymus-specific protease PRSS16 are differentially expressed in human thymus. *Genes Immun* 2005; **6:** 1-7.

36. Passerini L, Bernasconi P, Baggi F, Cornelio F, Mantegazza R: Analysis of SjTREC levels in thymus from MG patients and normal children. *Ann N Y Acad Sci* 2003; **998:** 270-274.

37. Helgeland G, Petzold A, Luckman SP, Gilhus NE, Plant GT, Romi FR: Matrix metalloproteinases in myasthenia gravis. *Eur Neurol* 2011; **65:** 53-58.

38. Romi FR, Gilhus NE, Luckman SP: Serum matrix metalloproteinase-3 levels are elevated in myasthenia gravis. *J Neuroimmunol* 2008; **195:** 96-99.

39. Suzuki S, Satoh T, Yasuoka H *et al*: Novel autoantibodies to a voltage-gated potassium channel Kv1.4 in a severe form of myasthenia gravis. *J Neuroimmunol* 2005; **170:** 141-149.

40. Ragheb S, Lisak R, Lewis R, Van Stavern G, Gonzales F, Simon K: A potential role for B-cell activating factor in the pathogenesis of autoimmune myasthenia gravis. *Arch Neurol* 2008; **65:** 1358-1362.

41. Moser B, Bekos C, Zimprich F, Nickl S, Klepetko W, Ankersmit J: The receptor for advanced glycation endproducts and its ligands in patients with myasthenia gravis. *Biochem Biophys Res Commun* 2012; **420:** 96-101.

42. Marinova TT, Velikova KK, Petrov DB *et al*: Structural and ultrastructural localization of NGF and NGF receptors in the thymus of subjects affected by myasthenia gravis. *Autoimmunity* 2004; **37:** 587-592.

43. Marinova TT, Kuerten S, Petrov DB, Angelov DN: Thymic epithelial cells of human patients affected by myasthenia gravis overexpress IGF-I immunoreactivity. *APMIS* 2008; **116:** 50-58.

44. Buckley C, Newsom-Davis J, Willcox N, Vincent A: Do titin and cytokine antibodies in MG patients predict thymoma or thymoma recurrence? *Neurology* 2001; **57:** 1579-1582.

45. Onodera J, Nakamura S, Nagano I *et al*: Upregulation of Bcl-2 protein in the myasthenic thymus. *Ann Neurol* 1996; **39:** 521-528.

46. Shiono H, Fujii Y, Okumura M, Takeuchi Y, Inoue M, Matsuda H: Failure to down-regulate Bcl-2 protein in thymic germinal center B cells in myasthenia gravis. *Eur J Immunol* 1997; **27:** 805-809.

47. Salakou S, Tsamandas AC, Bonikos DS, Papapetropoulos T, Dougenis D: The potential role of bcl-2, bax, and Ki67 expression in thymus of patients with myasthenia gravis, and their correlation with clinicopathologic parameters. *Eur J Cardiothorac Surg* 2001; **20:** 712-721.

48. Jander S, Stoll G: Increased serum levels of the interferon-gamma-inducing cytokine interleukin-18 in myasthenia gravis. *Neurology* 2002; **59:** 287-289.

49. Chuang WY, Strobel P, Gold R *et al*: A CTLA4high genotype is associated with myasthenia gravis in thymoma patients. *Ann Neurol* 2005; **58:** 644-648.

50. Wang XB, Pirskanen R, Giscombe R, Lefvert AK: Two SNPs in the promoter region of the CTLA-4 gene affect binding of transcription factors and are associated with human myasthenia gravis. *J Intern Med* 2008; **263:** 61-69.

51. Giraud M, Taubert R, Vandiedonck C *et al*: An IRF8-binding promoter variant and AIRE control CHRNA1 promiscuous expression in thymus. *Nature* 2007; **448:** 934-937.

52. Guyon T, Wakkach A, Poea S *et al*: Regulation of acetylcholine receptor gene expression in human myasthenia gravis muscles. Evidences for a compensatory mechanism triggered by receptor loss. *J Clin Invest* 1998; **102:** 249-263.

53. Giraud M, Eymard B, Tranchant C, Gajdos P, Garchon HJ: Association of the gene encoding the delta-subunit of the muscle acetylcholine receptor (CHRND) with acquired autoimmune myasthenia gravis. *Genes Immun* 2004; **5:** 80-83.

54. Viken MK, Sollid HD, Joner G *et al*: Polymorphisms in the cathepsin L2 (CTSL2) gene show association with type 1 diabetes and early-onset myasthenia gravis. *Hum Immunol* 2007; **68:** 748-755.

55. van der Pol WL, Jansen MD, Kuks JB *et al*: Association of the Fc gamma receptor IIA-R/R131 genotype with myasthenia gravis in Dutch patients. *J Neuroimmunol* 2003; **144:** 143-147.

56. Suhail H, Soundararajan CC, Vivekanandhan S, Singh S, Behari M: Apolipoprotein-E genotypes and myasthenia gravis. *Neurol India* 2010; **58:** 443-445.

57. Xu BY, Huang D, Pirskanen R, Lefvert AK: beta2-adrenergic receptor gene polymorphisms in myasthenia gravis (MG). *Clin Exp Immunol* 2000; **119:** 156-160.

58. Yilmaz V, Tutuncu Y, Baris Hasbal N *et al*: Polymorphisms of interferon-gamma, interleukin-10, and interleukin-12 genes in myasthenia gravis. *Hum Immunol* 2007; **68:** 544-549.

59. Alseth EH, Nakkestad HL, Aarseth J, Gilhus NE, Skeie GO: Interleukin-10 promoter polymorphisms in myasthenia gravis. *J Neuroimmunol* 2009; **210:** 63-66.

60. Sciacca FL, Ferri C, Veglia F *et al*: IL-1 genes in myasthenia gravis: IL-1A -889 polymorphism associated with sex and age of disease onset. *J Neuroimmunol* 2002; **122:** 94-99.

61. Pal Z, Antal P, Millinghoffer A *et al*: A novel galectin-1 and interleukin 2 receptor beta haplotype is associated with autoimmune myasthenia gravis. *J Neuroimmunol* 2010; **229:** 107-111.

62. Pal Z, Varga Z, Semsei A *et al*: Interleukin-4 receptor alpha polymorphisms in autoimmune myasthenia gravis in a Caucasian population. *Hum Immunol* 2012; **73:** 193-195.

63. Suzuki S, Utsugisawa K, Iwasa K *et al*: Autoimmunity to endoplasmic reticulum chaperone GRP94 in myasthenia gravis. *J Neuroimmunol* 2011; **237:** 87-92.

64. Du AL, Ren HM, Lu CZ, Tu JL, Xu CF, Sun YA: Carbonic anhydrase III is insufficient in muscles of myasthenia gravis patients. *Autoimmunity* 2009; **42:** 209-215.

65. Li X, Xiao BG, Xi JY, Lu CZ, Lu JH: Decrease of CD4(+)CD25(high)Foxp3(+) regulatory T cells and elevation of CD19(+)BAFF-R(+) B cells and soluble ICAM-1 in myasthenia gravis. *Clin Immunol* 2008; **126:** 180-188.

66. Gregersen PK, Kosoy R, Lee AT *et al*: Risk for myasthenia gravis maps to a (151) Pro-->Ala change in TNIP1 and to human leukocyte antigen-B*08. *Ann Neurol* 2012.

67. Zhang J, Chen Y, Jia G *et al*: FOXP3 -3279 and IVS9+459 polymorphisms are associated with genetic susceptibility to myasthenia gravis. *Neurosci Lett* 2012.
